# Ultrasound-activated erythrocyte membrane-camouflaged Pt (II) layered double hydroxide enhances PD-1 inhibitor efficacy in triple-negative breast cancer through cGAS-STING pathway-mediated immunogenic cell death

**DOI:** 10.7150/thno.102284

**Published:** 2025-01-02

**Authors:** Yanjie Wu, Zhiyu Zhao, Mengli Ma, Weijin Zhang, Wei Liu, Xiaochen Liang, Ting Zhao, Yi Luo, Yunjie Wang, Mengqi Li, Tingting Li, Cong Liu, Xian Luo, Shengyu Wang, Wanyun Li, Wei Zeng, Hong Wang, Wengang Li, Ting Wu, Zhihai Ke, Fanghong Luo

**Affiliations:** 1 Cancer Research Center, School of Medicine, Xiamen University, Xiamen, 361102, China.; 2 School of Science and Engineering, Shenzhen Key Laboratory of Innovative Drug Synthesis, The Chinese University of Hong Kong, Shenzhen, 518172, China.; 3 Thomas Lord Department of Mechanical Engineering and Materials Science, Duke University, Durham, NC, 27708, USA.; 4 The First Affiliated Hospital of Guangdong Pharmaceutical University, Guangdong Pharmaceutical University, Guangzhou, 510026, China.; 5 Environmental Toxicology, University of California, Riverside, CA, 92507, USA.; 6 Institute of Immunotherapy, School of Basic Medicine, Fujian Medical University, Fuzhou, 350122, China.; 7 Department of Gastroenterology, Department of Obstetrics and Gynecology, Affiliated Xiang'an Hospital, Medical Center, Xiamen University, 361000, China.; 8 Department of Anesthesiology, Shandong Provincial Hospital Affiliated to Shandong First Medical University, Jinan, Shandong, 250021, China.

**Keywords:** Layered double hydroxides (LDHs), Breast Cancer, Immunogenic cell death, Pyroptosis, Ferroptosis

## Abstract

**Rationale:** Immunogenic cell death (ICD) offers a promising avenue for the treatment of triple-negative breast cancer (TNBC). However, optimizing immune responses remains a formidable challenge. This study presents the design of RBCm@Pt-CoNi layered double hydroxide (RmPLH), an innovative sonosensitizer for sonodynamic therapy (SDT), aimed at enhancing the efficacy of programmed cell death protein 1 (PD-1) inhibitors by inducing robust ICD responses.

**Methods:** Pt-CoNi layered double hydroxide (LDH) nanocages were synthesized using a two-step method, followed by functionalization with red blood cell membranes to prepare RmPLH. The *in vitro* assessments included evaluations of cell toxicity, cellular uptake, and sonodynamic effects of RmPLH. Key mechanisms—such as oxidative stress, DNA damage, pyroptosis, cGAS/STING pathway activation, and inhibition of cellular migration and invasion—were explored under varying treatment conditions in 4T1 cells. Tumor-bearing mice were employed to evaluate tumor-targeting capabilities and the synergistic tumor-suppressive effects of RmPLH combined with PD-1 inhibitors. Comprehensive safety evaluations, including blood tests, biochemical analyses, and histopathological examinations, were also conducted.

**Results:** The synthesized Pt-CoNi LDH exhibited a uniform rhombic dodecahedral nanocage morphology with an average particle size of approximately 231 nm. Encapsulation with red blood cell membranes conferred prolonged systemic circulation, enhanced tumor targeting, and reduced immune clearance for RmPLH. Upon ultrasound (US) stimulation, the LDH released substantial levels of reactive oxygen species (ROS) and platinum ions. The ROS effectively induced endoplasmic reticulum stress and ferroptosis, while platinum ions facilitated DNA crosslinking, triggering significant DNA damage. ROS-induced pyroptosis released inflammatory mediators and damage-associated molecular patterns (DAMPs), which activated the cGAS/STING pathway and reinforced ICD. Combining RmPLH with PD-1 inhibitors significantly enhanced therapeutic efficacy against TNBC. Furthermore, safety assessments confirmed the excellent biocompatibility and biosafety of RmPLH**.**

**Conclusion:** The integration of RmPLH with PD-1 inhibitors substantially amplifies ICD, fostering robust antigen-specific T cell immunity and offering a promising therapeutic strategy for TNBC. This study represents a pioneering application of Pt (II)-based LDH nanocages in oncology, laying a foundation for future innovations in tumor immunotherapy.

## Introduction

Triple-negative breast Cancer (TNBC) is a subtype of breast cancer that lacks estrogen receptor (ER), progesterone receptor (PR), and human epidermal growth factor receptor 2 (HER2) 1. Due to the lack of these receptors, traditional endocrine therapy and HER2-targeted therapy are ineffective in TNBC, resulting in limited treatment options and poor prognosis 2. Red blood cell-encapsulated nanomedicine (RBC-NP) is an innovative drug delivery technology that combines the natural advantages of red blood cells with cutting-edge achievements in nanotechnology 3. Red blood cells are ideal drug carriers due to their long circulation time in the body and excellent biocompatibility 4. By encapsulating nanomedicines within the red blood cells, this technology can extend the residence time of drugs in the body, thereby enhancing their bioavailability and efficacy 5. Additionally, the protective effect of the red blood cell membrane prevents rapid degradation of drugs in the body, thereby improving their stability and effectiveness. This technology provides an efficient, safe, and flexible new approach to drug delivery, with broad application prospects in the fields of cancer therapy, anti-infection treatments, and gene therapy 6.

Programmed cell death protein 1 (PD-1) is a pivotal immune checkpoint that orchestrates immune responses in the body. Inhibiting PD-1 or its ligand PD-L1 can reinvigorate T-cell functionality, thereby enhancing the efficacy of the immune system against malignant cells 7. In recent years, considerable attention has been directed towards immunogenic cell death (ICD), due to its capacity to elicit tumor-specific adaptive immune responses 8. Various agents, including radiotherapy, chemotherapy, photodynamic therapy (PDT), and sonodynamic therapy (SDT), have emerged as significant inducers of ICD 9. These modalities provoke the release of tumor-associated antigens and danger-associated molecular patterns (DAMPs), consequently eliciting antigen-specific immune responses 10. Harnessing the ICD phenomenon via therapeutic interventions holds promise in transitioning "cold tumors" into "hot tumors," fostering an immunogenic tumor microenvironment that enhances antitumor immune responses. Such strategies serve as promising adjuncts to immunotherapy, ultimately augmenting patient response rates.

Layered double hydroxides (LDHs) have emerged as a fascinating class of materials for drug delivery and tumor treatment, offering remarkable physicochemical advantages such as excellent biocompatibility, acid-responsive biodegradability, and highly tunable chemical composition and structure, showing great potential for biomedical applications 11. Compared to bulk and nanosheet LDH, hollow LDH nanostructures provide advantages such as low mass density, high surface area, and maximized exposure to active sites 12. Owing to their nanoporous crystalline structure, metal organic frameworks (MOFs) can serve as templates for the design and synthesis of hollow nanostructured LDH materials through a template-mediated growth method while retaining the high porosity of MOFs 13. LDHs consisting of Co^2+^ and Ni^2+^ not only act as hollow open-structured scaffolds with high surface areas but also possess intrinsic reactive oxygen species (ROS)-catalytic properties 14. Various platinum (Pt) anticancer drugs have been widely used in cancer treatment 15. Pt cations can be incorporated into MOFs via a cation exchange process 16. However, the application of hollow nanostructured Pt-LDHs as sonosensitizers in SDT and cancer therapy has not yet been reported.

Pt (II) complexes are widely used in the treatment of tumors; however, they also have limitations such as high toxicity, side effects, poor tumor accumulation, and susceptibility to drug resistance. To exert their effects on the tumor microenvironment, Pt (IV) prodrugs must be reduced to therapeutically active Pt (II) 17. In this study, a sacrificial template method was employed to obtain LDH-based Pt (II) via ion exchange and template etching. Pt (II) ions can capture electrons to facilitate the separation of electron-hole pairs, resulting excellent catalytic properties and enabling the decomposition of H_2_O_2_ in tumor cells under ultrasound (US) irradiation to generate increased levels of ROS 18. Simultaneously, the activated Pt (II) ions can insert into mitochondrial DNA and interfere with dsDNA, thereby inducing ICD 19. This study is the first to directly apply LDH-based Pt (II) in tumor therapy.

To enhance the therapeutic efficacy against TNBC, we developed a novel drug delivery system termed RBCm@Pt-CoNi LDH (RmPLH). This system leverages the red blood cell encapsulation to extend drug residence time within the body. Under US stimulation, the newly designed LDH releases amounts of ROS and platinum ions. ROS stimulation activates endoplasmic reticulum stress and ferroptosis, while platinum ions effectively interact with DNA, causing DNA damage. Cellular pyroptosis triggered by ROS releases inflammatory substances, and the resulting molecular damage patterns activate the stimulator of interferon genes (STING) pathway, and effectively induce the immunogenic death. These related molecular damage patterns activated the STING pathway, effectively inducing immunogenic death. By combining RmPLH with PD-1 inhibitors, we observed a synergistic enhancement in the therapeutic efficacy against TNBC. Our approach holds promise for achieving complete tumor eradication, presenting a potentially transformative strategy for clinical management.

## Methods

***Materials:*** 2-methylimidazole (MIM, 99.0%), cobalt nitrate hexahydrate (Co(NO_3_)_2_·6H_2_O, 98.0%), nickel(II) nitrate hexahydrate ((Ni(NO_3_)_2_·6H_2_O, 98.0%), and sodium hexachloroplatinate(IV) hexahydrate (Na_2_PtCl_6_·6H_2_O, 99.0%) were purchased from Sigma-Aldrich. Hoechst 33342, ThiolTrace Violet, 2,7-dichlorofluorescein diacetate (DCFH-DA), and Liperfluo were purchased from Dojindo. BODIPYTM 581/591 C11, Tetraethylbenzimidazolylcarbocyanine iodide (JC-1), and Dulbecco's Modified Eagle medium (RPMI-1640) were purchased from Thermo Fisher Scientific. Cell Counting Kit-8 (CCK-8), CFDA SE Cell Proliferation and Cell Tracking Kit, Cell Cycle and Apoptosis Analysis Kit, Calcein-AM/PI Double Stain Kit, Rhod-2, AM, Cell Permeant, ER-Tracker Green, MitoTracker Green FM, and LysoTracker Green DND-26 were acquired from Yeasen, China. GSH and GSSG Assay Kits, Total Antioxidant Capacity Assay Kit using the ABTS technique, CellTiter-Lumi Luminescent 3D Cell Viability Assay Kit, BeyoClic EdU Cell Proliferation Kit with Alexa Fluor 647, Mitochondrial Permeability Transition Pore Assay Kit (MPTP Assay Kit), Mito-Tracker Deep Red FM, Intracellular Iron Colorimetric Assay Kit, and Lipid Peroxidation MDA Assay Kit were purchased from Beyotime, China. The anti-gamma γ-H2A.X, cGAS, STING, p-STING, TBK1, p-TBK1, p65, p-p65, IRF3, and p-IRF3 antibody were purchased from Abcam, and the GPX4, XBP1S, LC-3 antibody were obtained from Proteintech. BV510-anti-mouse CD45, PE/CY7-anti-mouse CD80, PE-anti-mouse CD86, APC-anti mouse CD11c, BV421-anti-mouse CD44, PE-CY7-anti-mouse CD26L, BV605-anti-mouse CD11b, BV421-anti-mouse Foxp3, PE-anti-mouse F4/80, FITC-anti-mouse CD3, APC-anti-mouse CD8a and PE-anti-mouse CD8a antibody were purchased from BioLegend. RMP1-14 and DiR were purchased from MCE.

***Synthesis of ZIF-67*.** Co(NO_3_)_2_·6H_2_O (0.3 mmol, 87.3 mg) was dissolved in methanol (4 mL), denoted as solution A. 2-Methylimidazole (1.8 mmol, 147.8 mg) was dissolved in methanol (4 mL), denoted as solution B. After sonication for 5 min, solution B was added to solution A. The mixture was allowed to stand for 3 h. The precipitated product was collected by centrifugation (8000 rpm) and washed three times with methanol.

***Synthesis of Pt-ZIF-67***. During the cation exchange process, 10 mg of ZIF-67 nanocrystals were dispersed in 5 mL of deionized water. Then 1.1 mg (2 mmol L-1) of sodium hexachloroplatinate (IV) hydrate (Na_2_PtCl_6_·6H_2_O) was dissolved in 1 mL of deionized water and slowly added to the ZIF-67 solution under stirring conditions. The reaction was quenched after 3 h by centrifuging the suspension. The Pt-ZIF-67 precipitate was collected and washed twice with deionized water and three times with ethanol. Pt-ZIF-67 nanocrystals were obtained by drying the precipitate in a vacuum oven overnight at 60 °C.

***Synthesis of Pt-CoNi LDH.*** First, 12 mg of Pt-ZIF-67 was dispersed in 20 mL of ethanol, designated as solution A. Then 20 mL of an ethanol solution containing 24 mg of Ni(NO_3_)_2_·6H_2_O was prepared and denoted as B. Solution B was quickly added to solution A. The resulting mixture was stirred at room temperature for 10 min, and then heated to 80 °C for 5 min. The product was centrifuged and washed several times with methanol and dried at 40 °C overnight.

***Erythrocyte Membrane Extraction*.** Erythrocyte membranes were isolated using differential centrifugation. Briefly, fresh blood was collected from BALB/c mice and centrifuged at 1000 × g for 5 min at 4 °C to remove the buffy coat and plasma. The resulting RBCs were washed twice with 1×PBS and then suspended in 0.25×PBS to induce lysis through hypotonic treatment. Subsequent centrifugation at 15,000 × g for 7 min removed hemoglobin and other cytoplasmic components, yielding a pink precipitate. The RBC membranes were then collected, and their protein concentrations were quantified using a BCA assay (Thermo Fisher Scientific Inc.).

***Synthesis of RmPLH*.** Erythrocyte membranes were prepared by sonicating the Erythrocyte membranes and Pt-CoNi LDH at a 1:4 ratio in a bath sonicator for 6 min. The resulting mixture was then sequentially extruded through polycarbonate membranes with pore sizes of 1 μm, and 400 nm (Whatman, UK) using an Avanti Polar Lipids mini extruder.

***Analysis and Characterization:*** A scanning electron microscope SEM (MAIA 3 LMH, Czech Republic) was used to study the morphology of the samples. High-resolution TEM (HRTEM) images were acquired usingan FEI Tecnai F20 microscope operating at 200 kV. High-angle annular dark-field scanning transmission electron microscopy (HAADF-STEM) and energy-dispersive X-ray spectroscopy (EDS) elemental mapping images were obtained at 200 kV by using a JEOL JEM-NeoAEM-200F TEM. X-ray diffraction (XRD) patterns were measured using a Rigaku SmartLab diffractometer with Cu Kα radiation. X-ray photoelectron spectroscopy (XPS) was performed using a PHI 5000 VersaProbe III system. Fourier transform infrared (FTIR) spectra were recorded on a Thermo Scientific (Nicolet iS10, USA) spectrometer. The hydration kinetic diameter (Dh) and zeta potential were determined using dynamic light scattering (DLS; Brookhaven Instruments Omni).

***Analysis of Cellular Uptake:*** 4T1 cells were initially cultured in a confocal dish until they reached 80% confluence. We primarily adsorb RBITC onto PLH through physical adsorption, followed by encapsulation with a red blood cell membrane. We take the required amount of RBITC at a ratio of 1mg PLH to 0.01mg RBITC, and dissolve RBITC in DMSO solution at a ratio of 1mg RBITC to 1mL DMSO. The dissolved RBITC solution is then added to the PLH solution and stirred continuously. Under the influence of physical adsorption forces, RBITC adsorbs onto PLH. After stirring for 24 hours, the liquid is collected and centrifuged, and thoroughly washed until the supernatant after centrifugation is colorless and transparent, leaving the PLH-RBITC precipitate for further use. Subsequently, the cells were treated with TRITC-labelled RmPLH to induce phagocytosis. The cells were analyzed at designated intervals throughout the incubation period. The phagocytic rate of RmPLH in 4T1 cells was assessed by nuclear staining with Hoechst 33342, followed by observation CLSM. Upon reaching 80% confluence in the confocal plate, 4T1 cells were exposed to TRITC-labeled RmPLH and allowed to co-localize for 12 Green fluorescent probes were employed to visualize and image various cellular organelles, including the ER, mitochondria, and lysosomes, for subsequent analysis of their co-localization. These probes ncluded ER-Tracker Green (BODIPY FL Glibenclamide) for ER visualization, Mito-Tracker Green (Benzoxazolium) for mitochondria, and LysoSensor Green DND-189 for lysosomal visualization. CLSM was utilized to quantify cellular uptake rates, while TEM was employed to investigate the intracellular localization and distribution of RmPLH within 4T1 cells. The 4T1 cells were seeded in 6-well plates at a density of 1×10^5^ cells/well and cultured for 24 h. Fresh medium containing PLH and RmPLH was added to the plates, and the cells were co-incubated for 1 and 4 h, respectively. After incubation, the excess medium was removed, and the cells were rinsed three times with ice-cold PBS. Subsequently, the cells were fixed in 4% paraformaldehyde and stained with DAPI for 30 min to enable laser confocal cell imaging. The cells were observed and photographed using CLSM at various time points.

***In vitro cytotoxicity evaluation:*** 4T1 cells were sourced from the American Type Culture Collection, and were cultured in high glucose RPMI-1640 supplemented with 1% penicillin-streptomycin and 10% fetal bovine serum (FBS). Cell viability was assessed using the CCK-8 assay (Yeason Biotechnology, China) following the manufacturer's instructions. Briefly, 96-well plates were seeded overnight with 5×10^3^ 4T1 cells. After treatment, the cells in the US group were subjected to US exposure at 1 W/cm^2^ for 2 min. Subsequently, cell viability was quantified by measuring optical density (OD) at 450 nm using a SpectraMax microplate reader(Model 680; Bio-Rad Laboratories, Inc, Tokyo, Japan). For subsequent experiments, 4T1 cells were seeded in 6-well plates and treated with RmPLH for 12h after reaching 80% confluence. Cell viability was further assessed using Calcein-AM/PI staining. Following RmPLH treatment, the cells were subjected to various interventions, including ultrasonic therapy(1 W/cm^2^, 2 min). After an additional 4-hour incubation period, cells were stained with Calcein-AM/PI for 25 min at room temperature in the dark, washed with PBS, and subsequently analyzed using CLSM. The concentration of RmPLH employed in these experiments was 20 μg/mL.

***3D cell culture:*** To minimize adsorption, cell culture was conducted using ultralow adsorption culture plates. Upon the formation of cellular spheres, various drug treatments were administered according to the assigned experimental groups. Following a 12-hour incubation period, images of the cellular spheres within each group were acquired, with the ultrasonic group subjected to US treatment at a previously determined power level. Subsequently, the cells were incubated for an additional 6 h, after which images were captured, and cell viability in the different treatment groups was assessed using the CellTiter-Lum Luminescence 3D Cell Viability Detection Kit.

***In vitro oxidative stress:*** Initially, 4T1 cells were seeded in a confocal dish and allowed to proliferate until reaching a density of 90%. Subsequently, various drugs were added according to the experimental design, and the cells were incubated for 12 h. Following this incubation period, US treatment was applied to the cells, followed by an additional 6-hour incubation. Intracellular levels of ROS, mitochondrial membrane potential, mitochondrial permeability transition pore (MPTP) opening, mitochondrial calcium ion concentration, and mitochondrial mass were assessed using fluorescent probes such as DCFH-DA and JC-1, following the protocols provided by the respective manufacturers. The nuclei were stained with Hoechst 33342. Finally, the results were analyzed using CLSM or flow cytometry techniques.

***Cell transwell analysis:*** Cells were cultured in an appropriate culture medium. Upon reaching the logarithmic growth phase, the cells were washed with PBS and digested with trypsin to prepare a single-cell suspension. The cell concentration was adjusted to 1×10^6^ cells/mL. For invasion assays, 100 µL of Matrigel was added to the upper layer of the Transwell chamber and incubated at 37 °C for 30 min to allow the Matrigel to solidify; this step was omitted for migration experiments. Subsequently, 200 µL of the serum-free cell suspension was added to the upper chamber of the Transwell apparatus, while 600 µL of complete culture medium containing 20% FBS was added to the lower chamber. The Transwell plate was then incubated at 37 °C with 5% CO_2_ for 24 h. After incubation, the Transwell chamber was removed, and the cells in the upper chamber were gently washed with PBS and fixed with 4% paraformaldehyde for 15 min. Following fixation, cells were stained with 0.1% crystal violet for 30 min. Non-invading cells in the upper chamber were gently removed using a cotton swab. The invasive cells on the membrane surface of the lower chamber were observed and counted using a microscope.

***Western blotting:*** Cell lysates were electrophoretically separated and subsequently transferred onto polyvinylidene difluoride (PVDF) membranes. Following overnight incubation at 4 °C with primary antibodies, membranes were subjected to blocking with Tris-buffered saline (TBS) supplemented with 5% skim milk. After three washes in TBS-Tween 20 (TBST) buffer, the membranes were incubated with secondary antibodies at room temperature for 45 min. Subsequently, the membranes were washed five times in TBS buffer containing 0.1% Tween-20 before images were captured using the ChemDoc Imaging system (Bio-Rad).

***Evaluation of IFN-β secretion level:*** 4T1 cells were seeded into 96-well plates at a density of 8×10^3^ cells per well and cultured in a constant temperature incubator at 37 °C with 5% CO_2_ for 24 h. Subsequently, the cells were cultured for an additional 12 h after undergoing various treatments. The release of IFN-β was quantified using the cytotoxicity assay kit as per the manufacturer's instructions.

***Immunofluorescence Staining:*** 4T1 cells were cultured in 12-well plates until reaching approximately 80% confluence. Subsequently, cells were allocated into distinct groups and administered either a placebo or medication. The US group received a 2-minute US therapy session using a machine operating at 1 W/cm². After treatment, the cells were fixed with 4% paraformaldehyde overnight, followed by permeabilization with 0.2% Triton X-100 on ice for 20 min. This was followed by three 10-minute rinses in 0.01 M phosphate-buffered saline (PBS). The cells were then blocked with 2% bovine serum albumin for 1 h at room temperature, rinsed three times with PBS (10 min each), and incubated overnight at 4 °C with rabbit anti-GPX4 (1:200; Abcam) and rabbit anti-H2A.X (1:100; Abcam) primary antibodies. The following day, the cells were treated with Alexa Fluor 488 donkey anti-rabbit antibody (1:1000; Abcam) at room temperature 1 h, followed by three washes with 0.01 M PBS. After fixation in a DAPI-containing medium and three additional PBS washes, the cells were subjected to CLSM for three-dimensional reconstruction.

***Detection of intracellular lipid oxidation and antioxidant capacity:*** 4T1 cells were seeded at a density of 6 × 10^5^ cells per well in 6-well dishes and treated with RmPLH at a concentration of 20 μg/mL for a duration of 12 h. Subsequently, the cells were subjected to US treatment using a machine operating at 1 W/cm2 for 2 min. Following an additional 6-hour incubation period, changes in intracellular antioxidant capacity were assessed usingthe GSH and GSSG Assay Kit as well as the Total Antioxidant Capacity Assay Kit employing the ABTS method, adhering to the guidelines provided by the reagent manufacturer. The degree of lipid peroxidation was evaluated using BODIPY C11 and Liperfluo fluorescent probes, and the mean fluorescence intensity after treatment was quantified using CLSM. Additionally, the degree of lipid peroxidation was quantitatively assessed using Lipid Peroxidation MDA Assay Kit.

***In Vivo Targeting and Imaging:*** To create a subcutaneous tumor model, female BALB/c mice (6-8 weeks old) were obtained from the Xiamen University Experimental Animal Centre. The mice received a subcutaneous injection of 4T1 cells (1×10^6^ cells in 100 μL). When the tumor volumes reached 100 mm^3^, the mice were intravenously injected with 20 mg/kg of DiR-labeled RmPLH. Following an esthesia, fluorescence imaging was performed at 1, 4, 8, 12, 24, 48, 72, 96, 120, and 144 h using a PerkinElmer IVIS Lumina III instrument. Tumors and major organs were harvested 144 h after the mice were implanted with fluorescent tags, and the animals were euthanized for ex vivo fluorescence imaging.

***The Efficacy of RmPLH for Cancer:*** To evaluate the anticancer effect of SDT on RmPLH, a subcutaneous tumor model was established in female BALB/c mice. Once the average tumor volume reached 100 mm³. The tumor-bearing mice were randomized into four treatment groups: (I) saline, (II) ultrasound (US), (III) RmPLH, and (IV) RmPLH + US. Each mouse received a tail vein injection of 100 μL of the assigned treatment, with RmPLH administered at a dosage of 20 mg/kg. 24 hours post-injection, the tumor sites in the respective group received US treatment at a frequency of 1.0 MHz, and an intensity of 1.5 W/cm², for 2 min. Throughout the experiment, the mice were weighed twice daily, and tumor dimensions (length [L] and width [W]) were measured using Vernier calipers and a microelectronic scale. The tumor volume (V) was calculated using the formula: V = L × W² / 2. After 14 days of treatment, tumor tissues and major organs were harvested from the euthanized mice. The harvested tumor tissues were subjected to H&E staining for further histopathological analysis.

***Preparation of Single-Cell Suspensions from Mouse Tissue and Flow Cytometry Analysis Procedure:*** First, the mice were euthanized, and the target tissues, such as the spleen or lymph nodes, were harvested. The tissue was placed in an ice-cold PBS buffer and scissors and forceps were used to mince the tissue. Next, the minced tissue was transferred in to a 50 mL centrifuge tube with a 200-mesh filter. The tissue was rinsed with PBS repeatedly until it was fully dispersed into a single-cell suspension. The single-cell suspension was collected into a new centrifuge tube and centrifuged at 300 × g for 5 min. The supernatant was discarded and the cell pellet was resuspended in PBS and centrifuged again. The resuspended cell suspension was passed through a 40 µm filter specific for flow cytometry to ensure that no large cell clumps or tissue fragments were remaining. Next, the cell suspension was treated with 1 mL of red blood cell lysis buffer to remove the red blood cells. The suspension was incubated at room temperature for 5 min after which 10 mL of PBS was immediately added to stop the reaction followed by centrifugation at 300 × g for 5 min. The supernatant was discarded, and the cell pellet was resuspended in PBS. These washing steps were repeated once more. For flow cytometry staining, 100 µL of resuspended cells were added to each flow tube, with approximately 1 ×10^6^ cells per tube. An appropriate amount of the antibody mixture was added to each flow tube and incubated on ice, protected from light, for 30 min. After staining, the cells were washed twice with PBS and centrifuged at 300 × g for 5 min. Finally, the cells were resuspended in an appropriate amount of flow cytometry analysis buffer and analyzed using a flow cytometer. Data collection and interpretation were performed using FlowJo software.

***In Vivo Biosafety Assessment:*** The obtained organs were fixed and H&E staining was applied to the tissue slices. Blood was drawn from each group, and standard blood analysis was performed.

All animal experiments were approved by the Xiamen University Ethics Committees (XMULAC20230200).

***Statistical Analysis:*** All values are expressed as mean ± SD, and the significance of the data is based on Student's t-test, one-way analysis of variance (ANOVA), and two-way ANOVA (*p < 0.05, **p < 0.01, ***p < 0.001 and ****p < 0.0001).

## Results and Discussion

### Synthesis and characterization of RmPLH

**Scheme 1:**
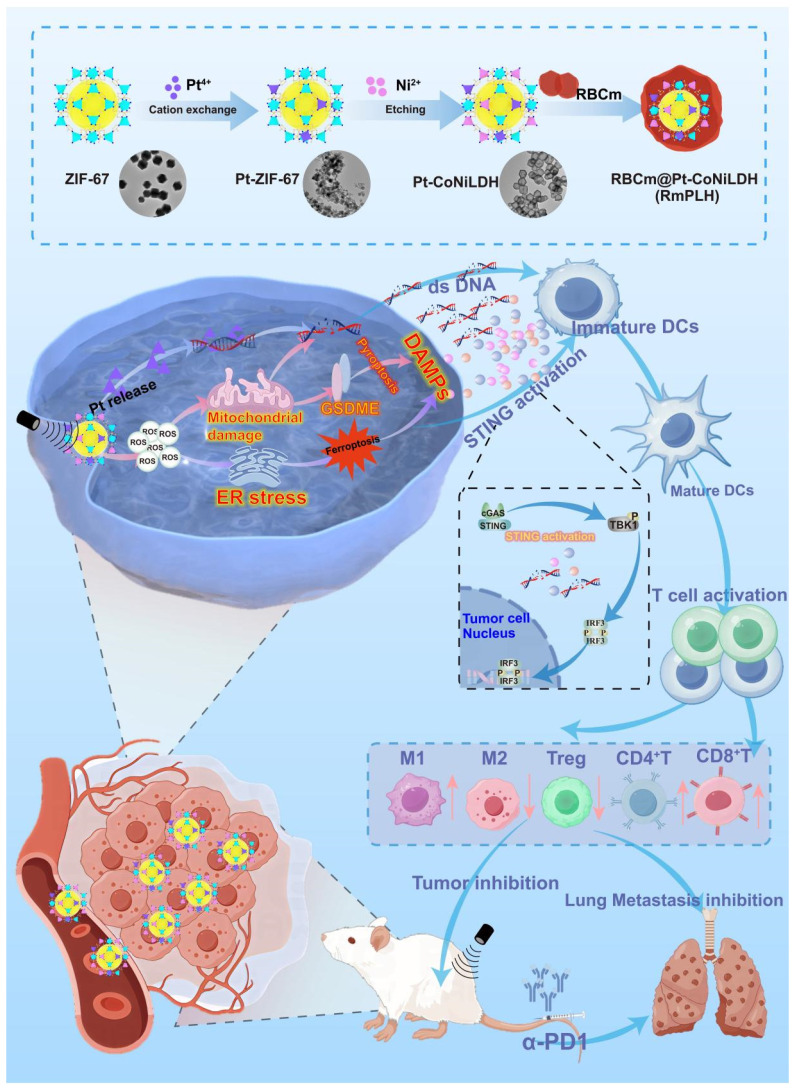
Ultrasound-Activated Erythrocyte Membrane-Camouflaged Pt(II)-LDH Enhances PD-1 Inhibitor Efficacy in Triple-Negative Breast Cancer through cGAS-STING Pathway-Mediated Immunogenic Cell Death.

**Figure 1:**
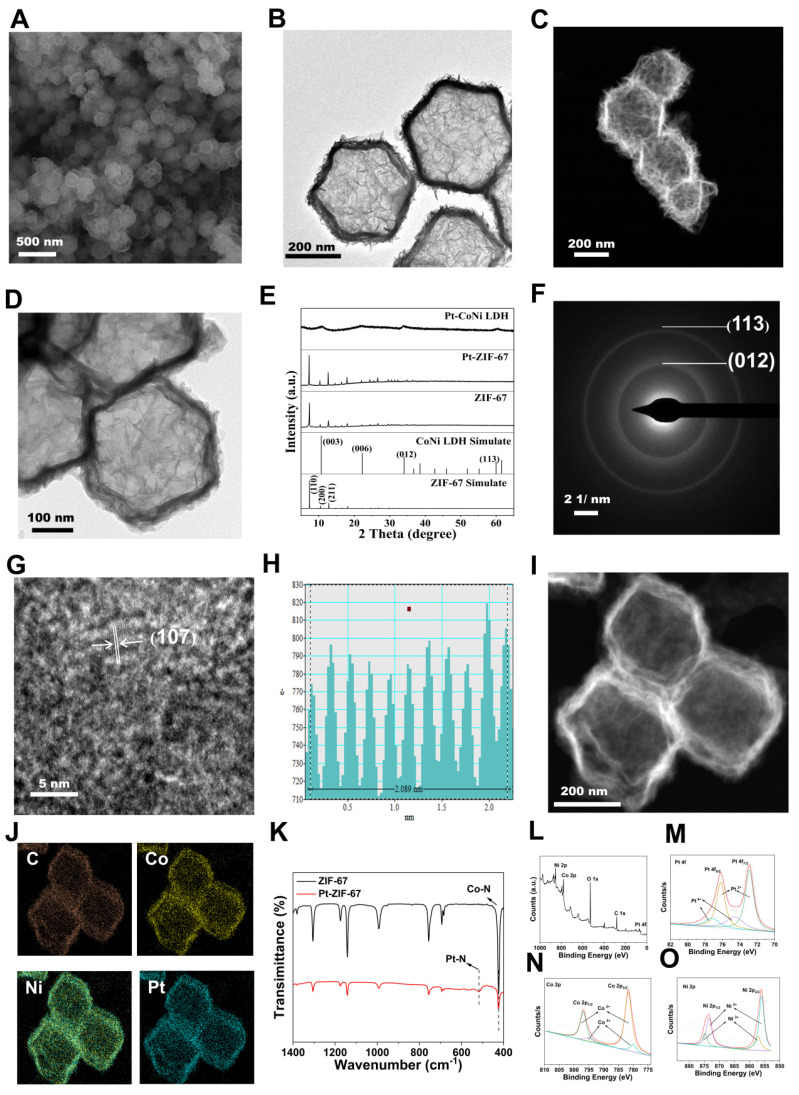
** Synthesis and characterization of RmPLH.** (A) SEM of Pt-CoNi LDH (the inset image is size distribution histogram); (B)TEM of Pt-CoNi LDH. (C) HAADF-STEM of Pt-CoNi LDH. (D) TEM of RmPLH.(E) XRD patterns of ZIF-67, Pt-ZIF-67 and Pt-CoNi LDH. (F) Selected area electron diffraction (SAED) pattern of Pt-CoNi LDH. (G) High resolution TEM (HRTEM) image of Pt-CoNi LDH. (H) Line-scan intensity profile from along with the direction of arrow of HRTEM image. (I) HAADF-STEM image of Pt-CoNi LDH and (J) Corresponding elemental mapping of C, Co, Ni and Pt elements. (K) FT-IR spectra of ZIF-67 and Pt-ZIF-67. (L) XPS survey scan spectra of Pt-CoNi LDH. (M) the Co 2p XPS spectrum of Pt-CoNi LDH. (N) The Ni 2p XPS spectrum of Pt-CoNi LDH. (O) The Pt 4f XPS spectrum of Pt-CoNi LDH.

The Pt-CoNi LDH was prepared using a two-step method (**Scheme 1**). Briefly, dodecahedral ZIF-67 powder was first synthesized (see Experimental Section for details) and dispersed in a solution of Na_2_PtCl_6_·6H_2_O. Pt-ZIF-67 was then synthesized under stirring conditions at room temperature for 3 h. The resulting Pt-ZIF-67 was then collected and re-dispersed in an ethanol solution of Ni(NO_3_)_2_·6H_2_O. Under hydrothermal conditions at 80 °C for 5 min, Pt-ZIF-67 was etched into a Pt-CoNi LDH nanocage structure, likely owing due to the hydrolysis process and the Kirkendall effect 20. As shown in Figure 1A, the scanning electron microscopy (SEM) image of Pt-CoNi LDH shows a characteristic dodecahedral appearance with uniform particle size. The particle diameters determined from dynamic light scattering (DLS) were approximately 231 nm (Figure S1). Transmission electron microscopy (TEM) images further revealed that Pt-CoNi LDH exhibits a uniform rhombic dodecahedral nanocage morphology (Figure 1B). As seen in Figure 1C, the surface of the hollow nanocage appears relatively rough as it is composed of many small nanosheets 21. The TEM image in Figure 1D confirms the successful encapsulation of Pt-CoNi LDH by the erythrocyte membrane. The diameter of RmPLH, as determined by DLS was approximately 252 nm, which is larger than the particle size of PLH (231nm), as shown in Figure S2. Additionally, zeta potential measurements showed that the zeta potential of PLH is positive, while that of RmPLH is negative (Figure S3). Furthermore, the SDS-PAGE of RmPLH matched that of the red cell membrane-derived vesicles, indicating that the red cell membrane proteins were retained in RmPLH (Figure S4). These results collectively confirm the successful coating of the erythrocyte membrane. The diffraction peaks of ZIF-67 located at 7.4^o^, 10.4^o^, 12.7^o^, 16.5^o^ and 18.1^o^, correspond to the (110), (200), (211), (013), and (222) planes, respectively, aligning with identical to those of the simulated ZIF-67 pattern (Figure 1E) 22. The XRD patterns of Pt-ZIF-67 and ZIF-67 show no significant changes, indicating that Pt incorporation has a negligible effect on the structure. The XRD patterns of Pt-CoNi LDH displayed peaks at 11.1°, 22.4°, 34.2°, and 60.7°, corresponding to the (003), (006), (012), and (113) planes, respectively, which were consistent with those of the simulated CoNi LDH results. The selected area electron diffraction (SAED) pattern of Pt-CoNi LDH revealed three diffraction rings corresponding to (200), (012) and (113) planes (Figure 1F). In the high-resolution TEM (HRTEM) image (Figure 1G), a lattice fringe with an interplanar spacing of 0.208 nm was observed (Figure 1H), which was attributed to the (107) crystal plane of Pt-CoNi LDH. Energy dispersive X-ray spectroscopy (EDX) elemental mapping of the Pt-CoNi LDH (Figure 1I-J) indicated the uniform distribution of C, Co, Ni and Pt elements. The atomic ratio of Pt, Co, and Ni in the Pt-CoNi LDH was approximately 1:21:3 (Figure S5). The band located at around 427 cm^-1^ corresponds to the Co-N band in ZIF-67, and an additional band at 520 cm^-1^ is ascribed to Pt-N in Pt-ZIF-67, indicating the successful incorporation of Pt to ZIF-67 through a cation exchange process (Figure 1K). XPS survey scan spectra of the Pt-CoNi LDH exhibited the presence of a Pt component in the Pt-CoNi LDH (Figure 1L)**.** The Co 2p X-ray photoelectron spectroscopy (XPS) spectrum of Pt-CoNi LDH further verified peaks at 801 and 784.2 eV, corresponding to the Co 2p_1/2_ and Co 2p_3/2_ signals of Co^2+^, and additional peaks at 798 and 782.5 eV, associated with Co 2p_1/2_ and Co 2p_3/2_ signals of Co^3+^ (Figure 1M). In the Ni 2p XPS spectra of Pt-CoNi LDH (Figure 1N), the binding energies around 873.7 and 855.9 eV can be attributed to Ni^2+^ 2p1/2 and Ni^2+^ 2p3/2, respectively. Besides, other main characteristic peaks at 857.3 and 874.8 eV reveal the existence of Ni^3+^ species in the Pt-CoNi LDH 23. These observations suggest that some Co^2+^/Ni^2+^ was oxidized to Co^3+^/Ni^3+^ by the dissolved O^2^ and NO^3-^ ions in the solution 24. The signals in Pt 4f XPS spectra of Pt-CoNi LDH at 74.6 and 77.2 eV (Figure 1O) were assigned to Pt^4+^, and the peaks at 72.7 eV and 76.2 eV correspond to Pt^2+^ species 25. This indicates that Pt^2+^ is the dominant Pt species in Pt-CoNi LDH, and the valence state changes after the ion exchange process, likely due to the redox reaction of Pt in the solution. DLS and ELS techniques, were employed to monitor changes in the particle size and zeta potential, assessing the stability of the nanoparticles in these environments. The results showed that the excellent stability of the RBCm@Pt-CoNi nanoparticles in these environments, with no significant changes in the particle size or zeta potential observed during the test period (Figure S6 and 7).

To further validate the release of Pt, an *in vitro* release model was established. We used simulated body fluid (PBS) as the release medium, and samples were collected at regular intervals. The concentration of Pt was determined using inductively coupled plasma mass spectrometry (ICP-MS). The release profile demonstrated an initial rapid release followed by a sustained slow release, likely attributable to the structurale and surface properties of the nanoparticles (Figure S8). These findings confirm the controlled release of Pt under ultrasonic action.

### *In vitro* sonodynamic therapy assessment and cell uptake

**Figure 2:**
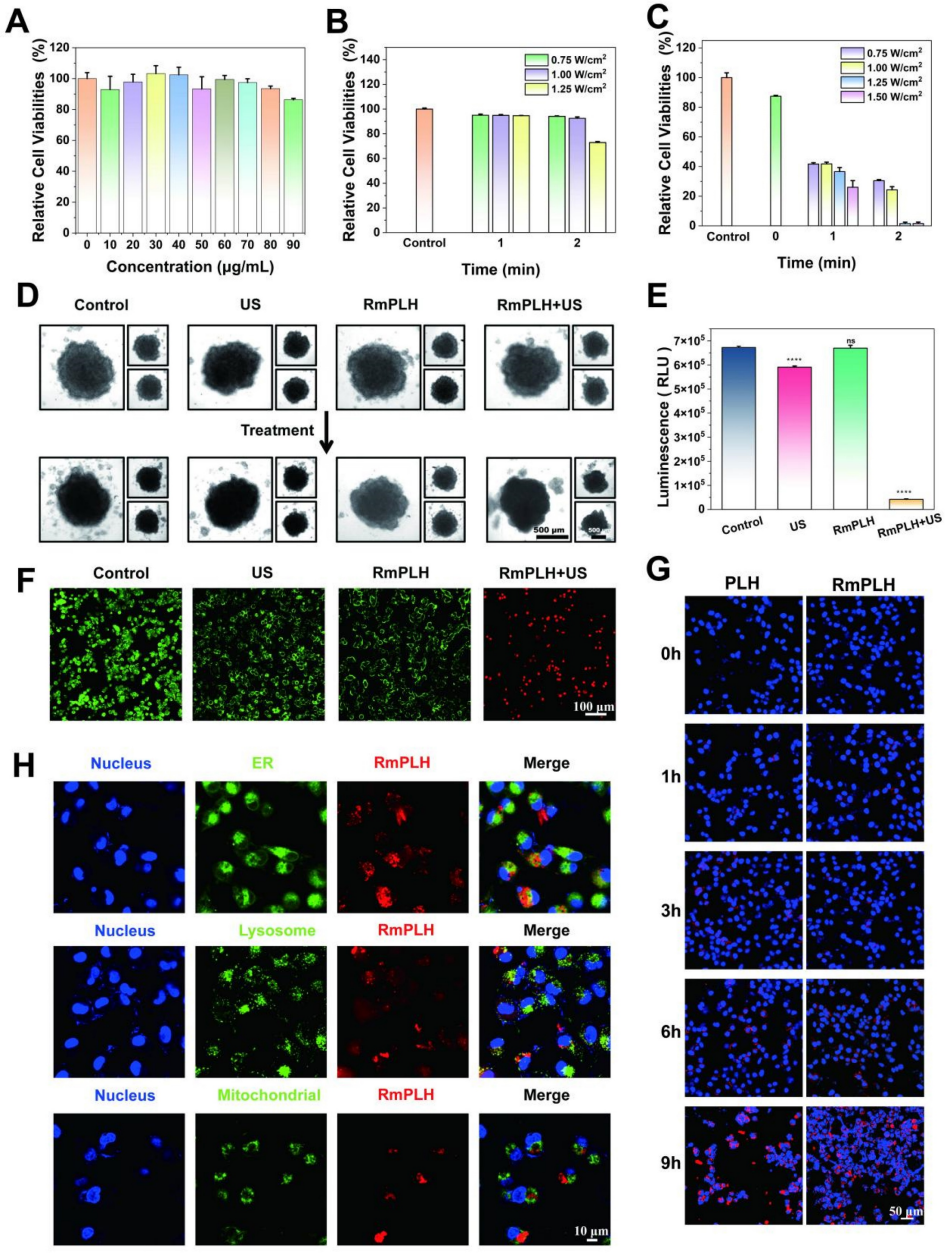
***In vitr*o sonodynamic therapy assessment and cell uptake.** (A) *In vitro* safety at different concentrations of RmPLH in 4T1 cells. (B)Relative viability of 4T1 cells under ultrasound alone (C) Relative viability of 4T1 cells with US under different power and time. (D) Morphology in a 3D tumor spheroid after different treatments. (E) ATP content in a 3D tumor spheroid after different treatments. (F) CLSM images of 4T1 stained with calcein-AM and PI followed with different treatments. (G) CLSM images of 4T1 cells incubated with PLH and RmPLH at different time points. (H) CLSM images of 4T1 cells after incubation with RBITC-labeled RmPLH for 12h, followed by ER Green, Mito-Tracker Green, and LysoTracker Green staining, respectively. Data were given as mean ± SD. ** *P* < 0.01, *** *P* < 0.001, n = 3.

The intracellular cytotoxicity of RmPLH was evaluated to assess its potential for biological applications. Incubation of 4T1 cells with varying concentrations of RmPLH revealed negligible cytotoxic effects even at concentrations of 90 μg/mL, with 4T1 cell viability remaining at 86.2%. This observation underscores the excellent biocompatibility of RmPLH (Figure 2A). Additionally, the impact of RmPLH combined with US irradiation on cell viability was investigated. First, the influence of US alone on cell viability was examined (Figure 2B). At power levels of 0.75 W/cm² and 1 W/cm² for 1 or 2 min, no significant alteration in cell viability was observed. However, at 1.25 W/cm² for 2 min, mechanical damage occurred, resulting in a 28% decrease in cell viability compared with the control group. Subsequently, the effect of different US powers and irradiation times on cell viability was assessed in the presence of RmPLH at a concentration of 20 μg/mL (Figure 2C). Based on these findings, it was determined that employing US parameters of 1 W/cm², 1.0 MHz power, and 2 minutes treatment duration would maintain cellular integrity and viability during subsequent experiments. Consequently, treatment groups were delineated into four categories: control, US, RmPLH, and RmPLH combined with US irradiation (1 W/cm², 2 minutes, 1.0 MHz), with RmPLH concentration set at 20 μg/mL. Notably, the RmPLH+US group exhibited conspicuous red fluorescence, indicative of enhanced cell-killing efficacy, compared with that of the control, US, and RmPLH-alone groups. To further elucidate the therapeutic efficacy of RmPLH in tumor tissues, 4T1 3D cell spheroids were cultured *in vitro*. Despite incomplete destruction of the spheres post-US irradiation in the RmPLH+US group, discernible alterations in the original spherical morphology were noted (Figure 2D)**.** Evaluation of overall cell viability using the CellTiter-Lum Luminescent 3D Cell Viability Assay Kit revealed a significant reduction in tumor cell spheroid viability following RmPLH treatment combined with US irradiation, with an average relative light unit (RLU) of 42089. In contrast, the RLUs exhibited by the control, US, and RmPLH groups were 672307, 590668, and 669536 respectively. These findings substantiate the potential of RmPLH as an effective sonosensitizer for SDT in cancer treatment (Figure 2E).

To differentiate between viable and non-viable cells post-treatment, a Calcein-AM/PI dual staining kit was used, and the samples were through confocal laser scanning microscopy (CLSM) (Figure 2F). To verify the effective cellular uptake of RmPLH, we pre-labeled PLH and RmPLH with RBITC. As depicted in Figure 2G, 4T1 cells incubated with RmPLH demonstrated increased red fluorescence intensities compared to those incubated with PLH at different time points. This indicates that RmPLH was taken up more efficiently than PLH and internalized by the cells. To further elucidate the intracellular localization of RmPLH following phagocytosis, fluorescent probes targeting the endoplasmic reticulum (ER), mitochondria, and lysosomes were employed 26. Analysis revealed a predominant distribution of RmPLH within the membrane system of 4T1 cells, particularly in the plasma, nuclear, and organelle membranes, while exclusion from the nucleus was observed. These results suggest that RmPLH targets the plasma membrane of 4T1 cells (Figure 2H). Collectively, these observations underscore the capacity of RmPLH to target breast cancer cells and its potential as a drug delivery system, while providing insights into its post-incorporation intracellular localization. Our findings may facilitate the development of more precise and effective drug delivery strategies for breast cancer therapy.

### *In vitro* oxidative stress effect of RmPLH under sonodynamic therapy

**Figure 3:**
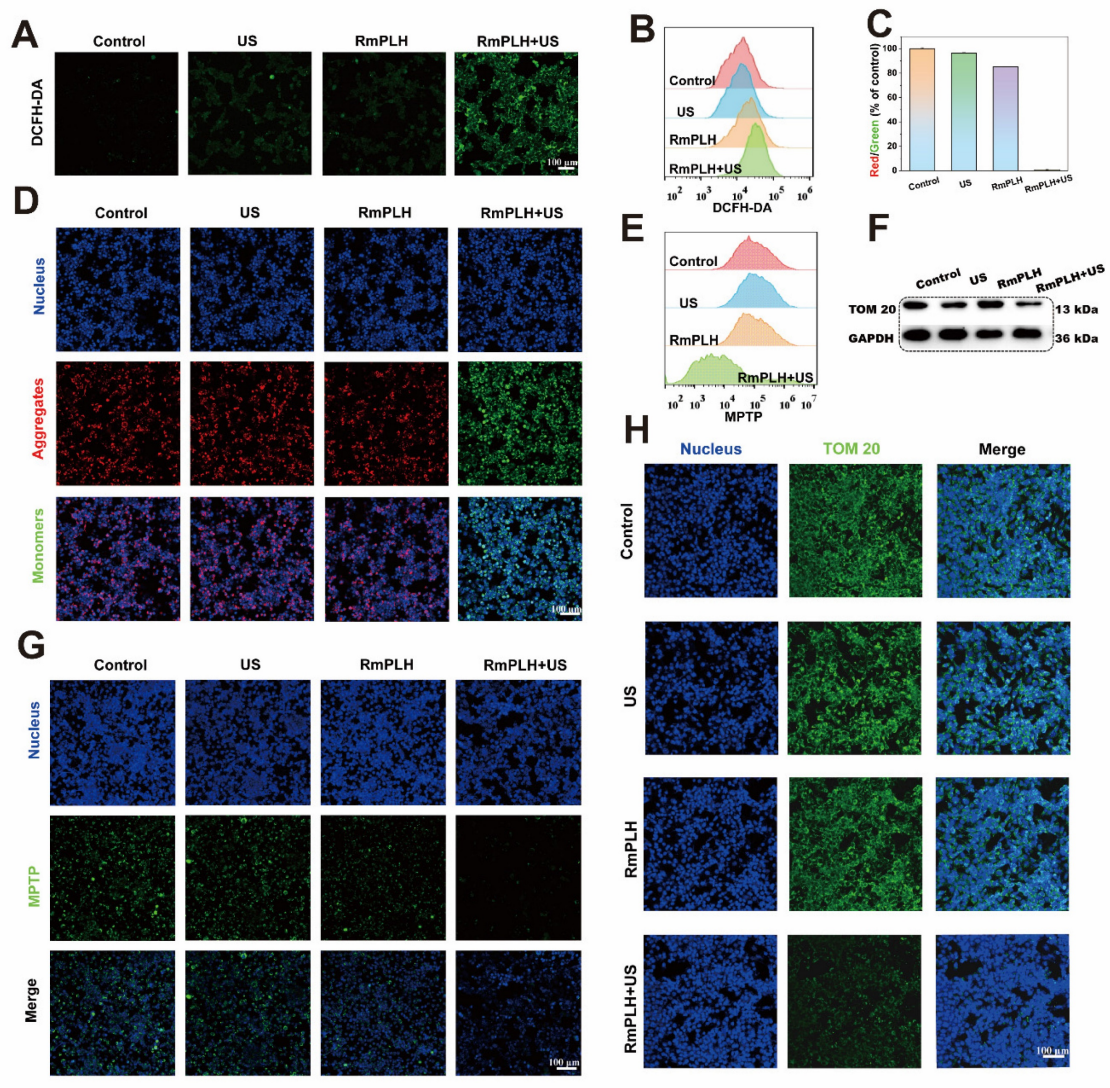
***In vitro* oxidative stress effect of RmPLH under sonodynamic therapy.** (A) Cellular CLSM images of DCFH-DA-stained 4T1 cells exposed to different treatments. (B) Flow cytometric analyses of DCFH-DA-stained 4T1 cells exposed to different treatments. (C) The appropriate red to green fluorescence intensity ratio of JC-1-stained 4T1 cells. (D) Cellular CLSM images of JC-1-stained 4T1 cells exposed to different treatments. (E) Flow cytometric analyses of MPTP-stained 4T1 cells exposed to different treatments. (F) Western blot of TOM 20. (G) Cellular CLSM images of MPTP-stained 4T1 cells exposed to different treatments. (H) Cell immunofluorescence of TOM 20. Data were given as mean ± SD. ** *P* < 0.01, *** *P* < 0.001, n = 3.

The potent cell-killing efficacy of RmPLH prompted the investigation into the underlying mechanisms under various conditions. To assess intracellular ROS levels, 2,7-dichlorofluorescein diacetate (DCFH-DA), a widely used ROS indicator, was employed 27. Upon oxidation, DCFH emits green fluorescence, which serves as an indicator of ROS production. As depicted in Figure 3A, negligible green fluorescence was observed in the control group, whereas weak fluorescence was evident in both the US and RmPLH groups. Conversely, the RmPLH+US group exhibited conspicuous green fluorescence, indicating substantial ROS generation upon exposure to the US. These findings were further corroborated by flow cytometry data (Figure 3B), which showed that tumor cells subjected to RmPLH+US treatment displayed the highest fluorescence intensity among all groups.

Mitochondria, pivotal cellular energy centers, are particularly vulnerable to ROS because of their membrane composition and internal milieu. Therefore, tetraethylbenzimidazolyl carbocyanine iodide (JC-1) staining was performed to evaluate mitochondrial membrane potential 28. Mitochondrial depolarization, indicative of potential disruption, manifests as increased green fluorescence owing to the presence of JC-1 monomers in the cytoplasm. As shown in Figure 3D, a significant increase in green fluorescence was observed in 4T1 cells treated with RmPLH following US irradiation, indicating pronounced mitochondrial depolarization. This observation was further substantiated quantitatively (Figure 3C), which showed that the ratio of red to green fluorescence markedly decreased after RmPLH + US treatment, indicated of US-induced mitochondrial depolarization. Opening of the mitochondrial permeability transition pore (MPTP) opening, which results in reduced mitochondrial membrane potential, causes mitochondrial dysfunction 29. To assess MPTP status in 4T1 cells post-treatment, an MPTP detection kit was used. The results revealed heightened mitochondrial membrane potential in the control, US, and RmPLH groups, as evidenced by strong green fluorescence within the mitochondria despite cytoplasmic fluorescence quenching by CoCl_2_ (Figure 3G). Conversely, in the RmPLH+US group, CoCl_2_ effectively quenched mitochondrial fluorescence, indicating of MPTP opening and severe mitochondrial dysfunction following RmPLH activation by US irradiation. Flow cytometry (Figure 3E) further corroborated these findings. TOM20 is an outer mitochondrial membrane protein and a key component of mitochondrial protein import and transport. The main function of TOM20 is to recognize the targeting peptide sequences on newly synthesized mitochondrial precursor proteins and help these proteins cross the outer mitochondrial membrane into the mitochondria 30. The impairment of mitochondrial function may affect the expression of TOM20. When mitochondrial membrane potential (MMP) is reduced, mitochondrial permeability increases, which may lead to the downregulation of TOM20 expression, thus affecting mitochondrial protein import and the normal function of the cell 31. Therefore, TOM20 expression was used to assess mitochondrial function. Immunofluorescence and western blotting analyses were used to assess TOM20 expression in 4T1 cells post-treatment. TOM20 protein expression was significantly diminished in the RmPLH+US group, indicating that US-activated RmPLH induces oxidative stress within cells, thereby affecting mitochondrial function (Figure 3F-H).

### Ferroptosis and pyroptosis *in vitro*

The augmented antioxidant capacity of cells has been associated with enhanced DNA repair capability, potentially contributing to drug resistance and therapeutic failure. Consequently, this study aimed to examine the antioxidant capacity and cell death mechanisms in 4T1 cells.

**Figure 4:**
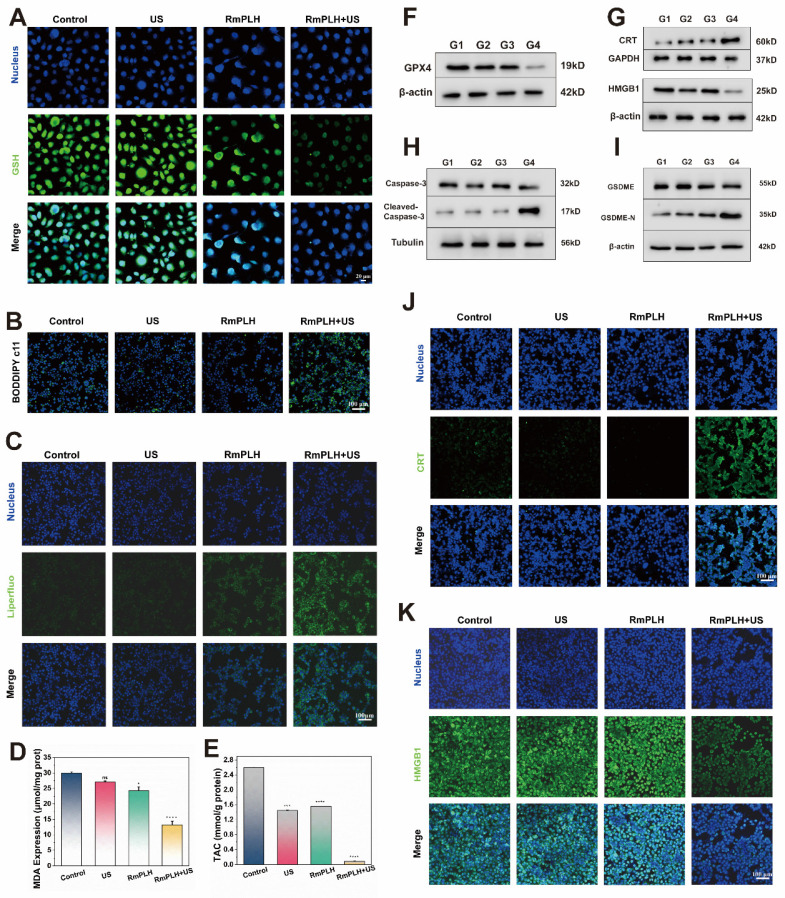
** Ferroptosis and pyroptosis *in vitro*.** (A) CLSM images of intracellular GSH in 4T1 cells treated with various treatments. ThiolTrace Violet discolored the GSH level (green). (B and C) CLSM images of 4T1 cells that were stained with BODIPY and Liperfluo following different treatments, respectively. (D) Assessment of total antioxidant capacity in 4T1 cells treated in the different groups. (E) TAC content in 4T1 cells treated with various treatments. (F) GPX4 protein western blot results. (G) HMGB1 and CRT protein western blot results. (H) caspase-3 and cleaved-caspase-3 protein western blot results. (I) GSDME and GSDME-N protein western blot results. (J) The immunofluorescence of the CRT protein in 4T1 cells following various therapies. (K) The immunofluorescence of the HMGB1 protein in 4T1 cells following various therapies. (G1: PBS, G2:US, G3: RmPLH, G4: RmPLH+US). Data were given as mean ± SD. ** *P* < 0.01, *** *P* < 0.001, n = 3.

Reduced glutathione (GSH) is a representative intracellular antioxidant whose, with its content reflects the robustness of the intracellular antioxidant system 32. As shown in Figure 4A, ThiolTracker Violet staining revealed robust green fluorescence in cells from the control and US-treated groups, indicating substantial intracellular GSH levels. Conversely, the RmPLH-treated group exhibited diminished fluorescence intensity, reflecting GSH depletion due to its utilization in cellular antioxidative processes. Glutathione Peroxidase 4 (GPX4), an essential antioxidant enzyme, plays a pivotal role in cellular homeostasis. GPX4 catalyzes the reduction of lipid peroxides into their corresponding alcohols, thereby safeguarding cell membrane integrity and preventing oxidative stress-induced cell death 33. As illustrated in Figure 4F, the protein expression of GPX4 was notably reduced in the RmPLH+US group. Previous studies have implicated the loss or dysfunction of GPX4 in rendering cells incapable of effectively clearing peroxidized lipids, consequently precipitating ferroptosis. Thus, we speculated that whether ferroptosis occurred in these cells based on the observed alterations in GPX4 expression. Subsequent analyses evaluated changes in intracellular lipids, lipid peroxides, and the metabolite of lipid peroxides, malondialdehyde (MDA), across the different treatment groups. BODIPY C11 staining (Figure 4B) and LiperFluo fluorescence (Figure 4C) revealed significantly increased fluorescence intensity in the RmPLH+US irradiation group, indicative of increased intracellular lipid peroxide accumulation. Moreover, a quantitative assessment of MDA content (Figure 4D) further substantiated the elevated intracellular lipid peroxide levels in the RmPLH+US group. Furthermore, as shown in Figure 4E, the ABTS method was employed to quantitatively determine the total antioxidant capacity of the cells following GSH reduction. Notably, the RmPLH+US group exhibited a significant decline in antioxidant capacity compared to the control group, as evidenced by the reduced MDA content. Collectively, these findings suggest substantial intracellular lipid peroxide accumulation as a consequence of GSH depletion and GPX4 inactivation, suggesting the induction of ferroptosis in 4T1 cells following RmPLH+US treatment. Ferroptosis is characterized by morphological changes in mitochondria including a smaller, rounder shape and the disappearance of the orderly arrangement of mitochondrial cristae, as shown by the blue arrow in Figure S9.

ROS play a key role in cell death, especially during pyroptosis. Pyroptosis is a form of programmed cell death that is often associated with an inflammatory response and is characterized by the rupture of the cell membrane and the release of cell contents, which triggers an inflammatory response 34. In RmPLH+ US-treated 4T1 cells, we observed significant ROS production, which is closely related to the activation of pyroptosis. The accumulation of ROS can lead to dysfunction of mitochondria and endoplasmic reticulum (ER), which in turn activates pyroptosis related signaling pathways. The high mobility group box 1(HMGB1) and calreticulin (CRT) are two key molecules released during thermonucleosis, playing essential roles in triggering the subsequent inflammatory response characterizing thermonuclear degenerative cell death. To investigate this, we examined the expression of HMGB1 and CRT proteins in cells from different treatment groups using cellular immunofluorescence and Western blotting (Figure 4G, J-K). Notably, HMGB1 protein expression was significantly down regulated, while CRT protein expression was significantly up-regulated in the RmPLH+US group compared to the control, US and RmPLH groups. In addition, the levels of the pyroptosis marker proteins Caspase-3 and gasdermin E (GSDME) were markedly reduced and significantly sheared in 4T1 cells from the RmPLH+US group (Figure 4H-I). The TEM result showed that 4T1 cells treated with RmPLH+US exhibited features typical of pyroptosis, which include the formation of a large number of vesicles (Figure S10). CLSM showed that 4T1 cells treated with RmPLH+US formed a large number of vesicles (Figure S11). These findings suggest that significant thermal apoptosis occurred in the 4T1 cells following treatment with RmPLH+US.

### cGAS/STING pathway activation

**Figure 5:**
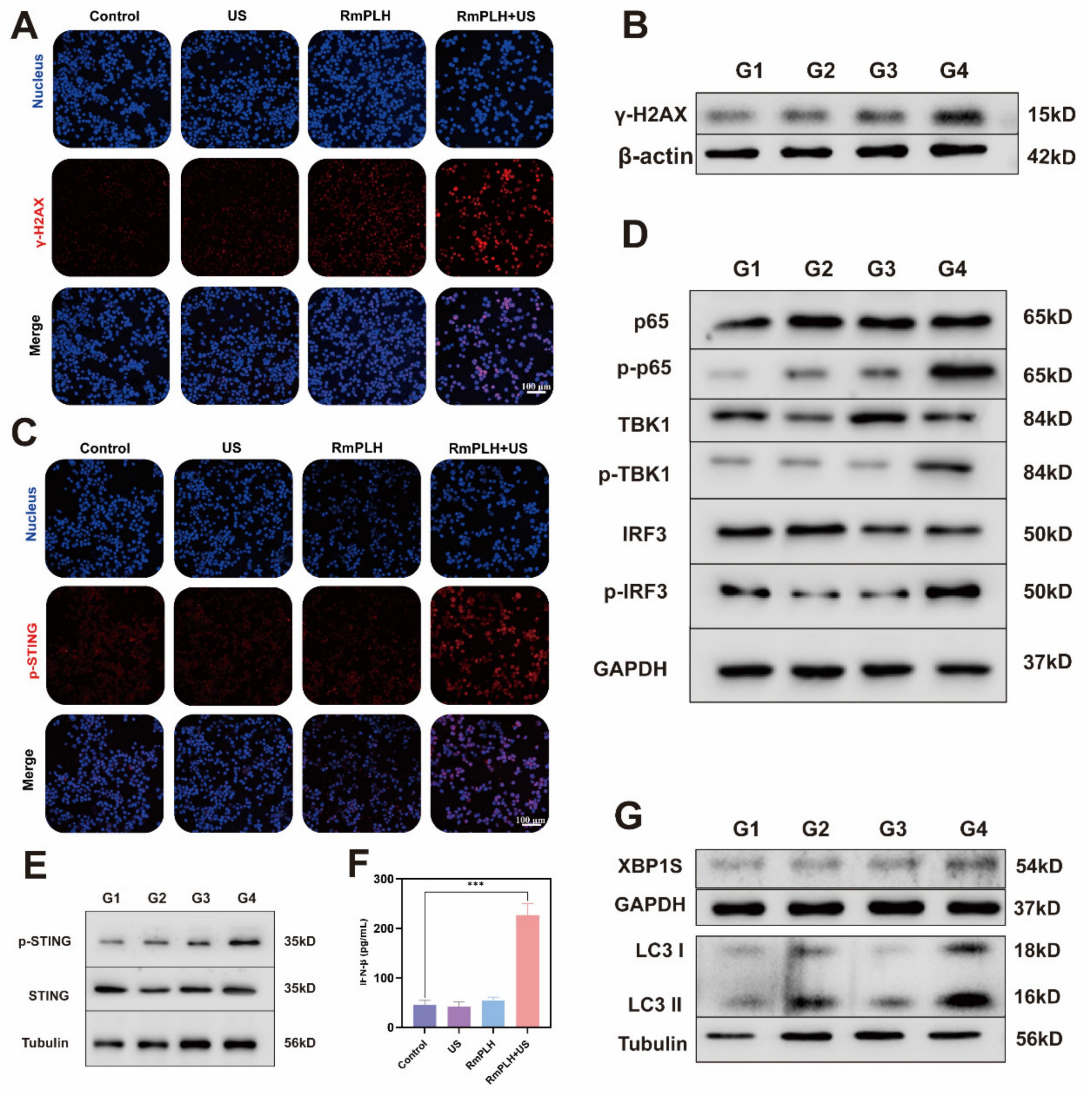
** cGAS/STING pathway activation.** (A) The immunofluorescence of the γ-H2A.X protein in 4T1 cells following various therapies. (B) Western blot results of γ-H2AX in 4T1 cells following various therapies. (C) The immunofluorescence of the p-STING protein in 4T1 cells following various therapies. (D and E) cGAS/STING pathway associated marker protein western blot results. (F) IFN-β expression in 4T1 cells under different treatments. (G) Endoplasmic reticulum stress and autophagy associated marker protein western blot results. (G1: PBS, G2:US, G3: RmPLH, G4: RmPLH+US). Data were given as mean ± SD. ** P < 0.01, *** P < 0.001, n = 3.

Drugs containing platinum ions chemically interact with tumor cell DNA, forming DNA adducts that disrupt the normal structure and function of DNA, thereby inducing cellular DNA damage 35. RmPLH, possessing a Pt structure akin to other platinum-based drugs, is hypothesized to induce significant intracellular DNA damage upon activation by US. This is evidenced by the occurrence of γ-H2A.X, a marker of intracellular DNA double-strand breaks and severe mitochondrial damage (Figure 5A-B). Figure 5C, demonstrates enhanced p-STING protein activation in cells treated with RmPLH+US treatment, corroborating this hypothesis. Additionally, Figure 5D and F highlight increased expression of cGAS/STING pathway-related proteins, including phosphorylated p65, phosphorylated TBK1, phosphorylated IRF3 and phosphorylated STING, further confirming the cGAS/STING pathway activation. Subsequently, the expression of IFN-β in 4T1 cells after different treatments was assessed using an ELISA kit. Results revealed that the treatment of RmPLH+US could induce IFN-β expression (Figure 5E).

Notably, intracellular mitochondrial and DNA damage have been implicated in calcium-ER stress coupling, leading to ER dysfunction and subsequent ER stress 36. As a pivotal player in cellular responses to ER stress, XBP1 pre-mRNA is activated and cleaved to form active the XBP1s protein, essential for maintaining cellular homeostasis and adapting to environmental changes 37. The increased intracellular expression of XBP1 following. In some studies, endoplasmic reticulum stress has been shown to promote the translocation of STING from the endoplasmic reticulum to the Golgi apparatus, a process that may involve ubiquitination of STING and interaction with endoplasmic reticulum membranes 38. In addition, endoplasmic reticulum stress may also activate the STING pathway by affecting mitochondrial function and the release of mtDNA. mtDNA, as a damage-associated molecular pattern (DAMP), can activate the cGAS, which in turn produces cGAMP, a direct activator of STING 39. The increased intracellular expression of XBP1 following RmPLH activation by US, underscores the induction of ER stress. Consequently, RmPLH + US treatment effectively instigates ER stress in cells, as indicated by increased levels of the autophagy-related marker protein, LC3 II, following ER stress induction (Figure 5G).

### *In vitro* migration and invasion ability

Activation of the cGAS/STING pathway exerts significant effects on the migratory and invasive properties of tumor cells by modulating various cellular processes, including epigenetic regulation, cell cycle dynamics, and apoptosis 40. Phalloidin, a cyclic heptapeptide toxin isolated from *Amanita phalloides*, exhibits a high binding affinity for filamentous actin (F-actin), with a dissociation constant (Kd) of 20 nM.

**Figure 6:**
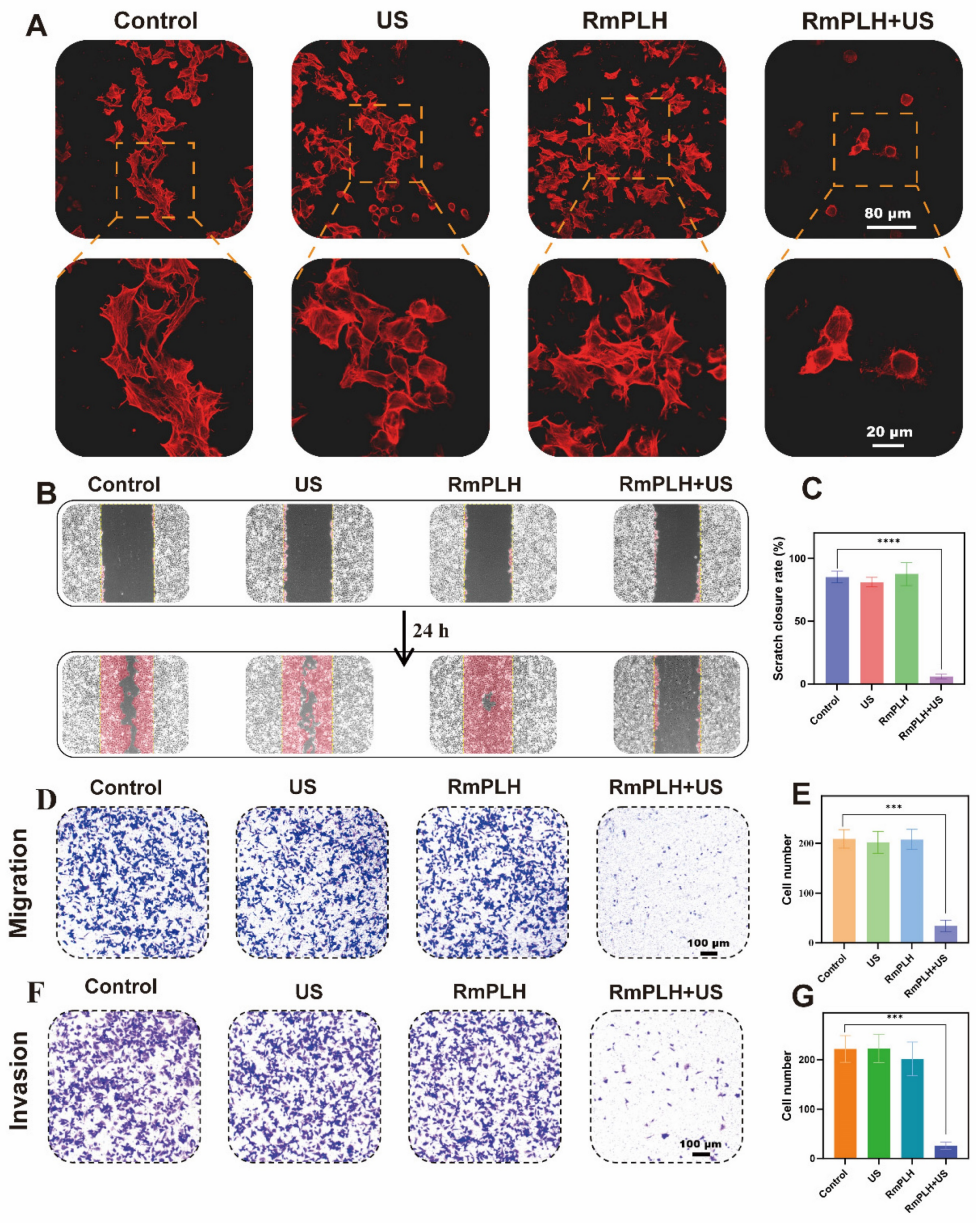
***In vitro* migration and invasion ability.** (A) TRITC Phalloidin staining in different treatment groups. (B) Cell wound healing experiment in different treatment groups. (C) Statistical analysis of cell wound healing experiment in different treatment groups. (D) Transwell cell migration experiment in different treatment groups. (E) Statistical analysis transwell cell migration experiment in different treatment groups. (F) Transwell cell invasion experiment in different treatment groups. (G) Statistical analysis transwell cell invasion experiment in different treatment groups. Data were given as mean ± SD. ** P < 0.01, *** P < 0.001, n = 3.

The experimental observations depicted in Figure 6A illustrate the distinct cellular morphologies across the different treatment groups. Specifically, the control, US-treated, and RmPLH-treated groups displayed a plethora of dynamic cellular structures characterized by protrusive, contractile, and stretchable microfilaments along with pseudopodial extensions on the cell surface. In contrast, the RmPLH+US-treated group exhibited augmented cytoplasmic volume, concomitant with a conspicuous absence of discernible pseudopodia.

Pseudopodial extensions play a pivotal role in facilitating tumor cell motility, localization, and phagocytic activity, all of which are closely linked to their invasive and metastatic potential 41. Subsequent assessment through a cell wound healing assay (Figure 6B-C) revealed that following a 24-hour post-treatment interval, cells in the control, US, and RmPLH groups exhibited migratory behavior towards the wound area, whereas migration was markedly attenuated in the RmPLH+US group.

Further assessment using the Transwell cell migration assay (Figure 6D-E**)** demonstrated a significant reduction in the number of cells traversing the porous membrane in the RmPLH+US group compared to that in the control, US, and RmPLH groups. Moreover, cell invasiveness, which denotes the ability of cells to penetrate their microenvironment, was evaluated using a Transwell cell invasion assay (Figure 6F-G). Cells in the control, US, and RmPLH groups displayed notable penetration through the Matrigel into the lower chamber, whereas a diminished number of cells in the RmPLH+US group exhibited the ability to penetrate the Matrigel barrier. Furthermore, the expression of Cyclin-d1 after different treatments was detected by WB, which further indicated that the treatment inhibited the migration and invasion of tumor cells **(**Figure S12**).** Collectively, these findings underscore the impact of RmPLH activation by US stimulation on the cGAS/STING pathway, leading to the consequential attenuation of the migration and invasion capabilities of 4T1 cells.

### The efficacy of RmPLH for cancer

**Figure 7:**
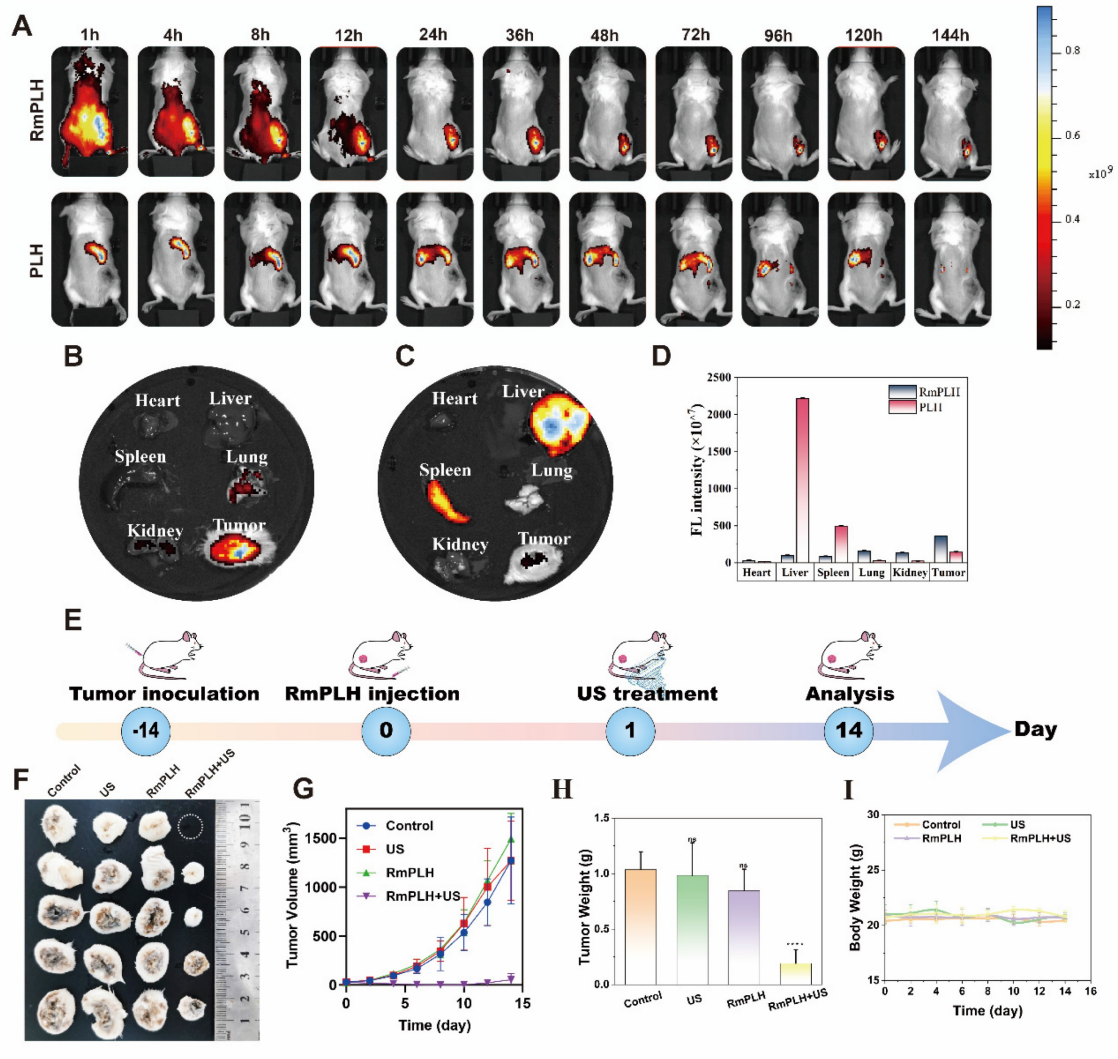
** The efficacy of RmPLH for cancer.** (A) *In vivo* fluorescence images of both PLH and RmPLH after intravenous injection of 4T1 tumor-bearing mice. (B)Fluorescence imaging of major organs and tumor tissues in the RmPLH group. (C) Fluorescence imaging of major organs and tumor tissues in the PLH group.(D) Quantitative fluorescence analysis of major organs and tumor tissues in RmPLH and PLH groups.(E) Schematic depiction of the treatment plan for SDT in the 4T1 tumor model mediated by RmPLH. (F) Digital photo images of tumors in various groups on the 14th day. (G) Growth curves of tumors in different groups. (H) The weight of tumors at the end of treatment. (I) Body weight changes of the mice under different treatments. Data were given as mean ± SD. ** P < 0.01, *** P < 0.001, n = 3.

The efficacy of our strategy in eradicating pre-existing tumors was assessed in BALB/c mice bearing 4T1 tumors. RmPLH demonstrated excellent tumor enrichment through the encapsulation of surface erythrocyte membranes. This was verified by *in vivo* imaging (Figure 7A). After injection RmPLH demonstrated the ability to target primary tumors 4 h post-injection and showed moderate fluorescence intensity after 48h. In contrast, stronger fluorescence signals were detected in other organs outside the tumor tissue in the PLH group. The *in vitro* organ imaging results were consistent with the *in vivo* observations, further confirming the effective enrichment of RmPLH (Figure 7B-D). Subsequently, tumor-bearing mice underwent one treatment followed by a 14-day monitoring period (Figure 7E). Tumor volumes exhibited steady increases following treatments with PBS, US, or RmPLH alone; in contrast, tumor volumes were significantly suppressed in the RmPLH+US group (Figure 7F-H, Table. S1). Although weight loss was observed in mice subjected to US treatment (Figure 7I), the subsequent recovery of body weight and histological examination via hematoxylin and eosin (H&E) staining of major organs (Figure S13) revealed no discernible damage to other organs or tissues, thereby establishing an acceptable safety profile. Routine blood tests were performed on all mice, and all results were within the safe range (Table S2). In our study, we procured tumor tissues from 4T1 tumor-bearing mice for subsequent immunohistochemical and immunohistofluorescent analysis. Specifically, the expression of the ferroptosis biomarker GPX4 was notably reduced in the RmPLH+US treatment group. Concurrently, there was a marked increase in the expression of GSDME-N, a pyroptosis-specific marker, and an elevation in the levels of the DNA damage marker γ-H2AX. Furthermore, the ICD markers CRT and HMGB1 exhibited divergent expression patterns, with CRT being upregulated and HMGB1 being downregulated. Additionally, an increase in the phosphorylation of STING was observed, suggesting the effective activation of the STING signaling pathway *in vivo*. (Figure S16). These findings underscore the complex interplay of cell death mechanisms and their potential implications for immunogenicity within the tumor microenvironment. Notably, the introduction of RmPLH coupled with US irradiation resulted in the complete inhibition of tumor growth, which was attributable to the activation of RmPLH by US, as corroborated by H&E staining and Ki-67 immunohistochemistry (Figure S14).

### Immunotherapy *via* the PD-1 and RmPLH

**Figure 8:**
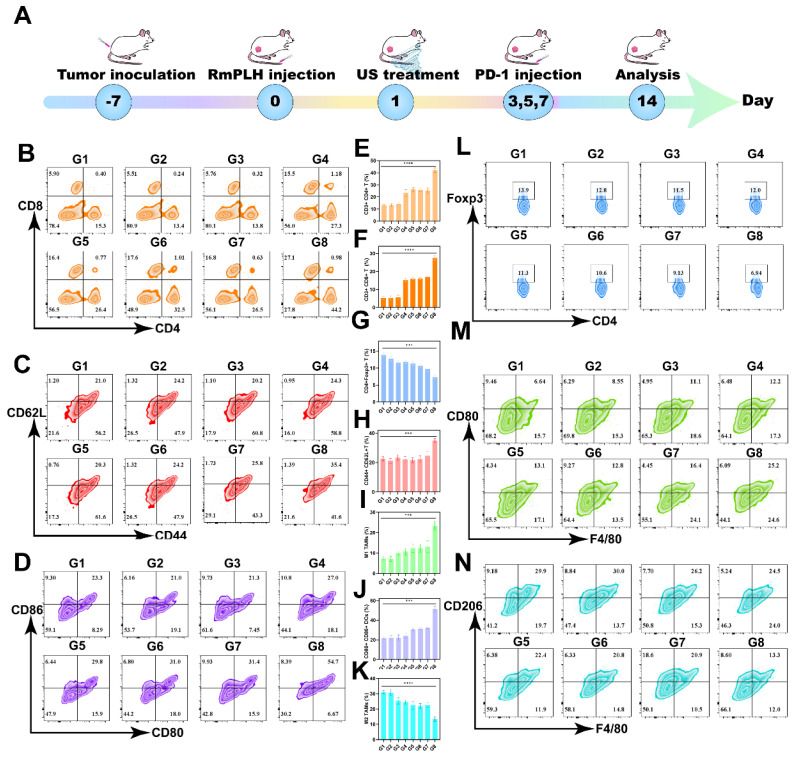
** Immunotherapy *via* the PD-1 and RmPLH.** (G1:PBS, G2:US, G3: PD-1, G4: PD-1+US, G5: PD-1+RmPLH, G6:PLH+US, G7:RmPLH+US,G8:PD-1+RmPLH+US) (A) Schematic depiction of the treatment plan for immunotherapy via the PD-1and RmPLH.(B) Flow cytometry to detect T cell activation in tumor cells. (C) Flow cytometry detection of effector T cell activation status at the TDLN. (D) Flow cytometry detection of the activation level of DCs at the TDLN. (E) Quantitative analysis of CD4+ T cells in tumor cells. (F) Quantitative analysis of CD8+ T cells in tumor cells. (G) Quantitative analysis of Tregs in tumor. (H) Quantitative analysis of effector T cell in TDLN. (I) Quantitative analysis of M1 in tumor. (J) Quantitative analysis of mature DCs in TDLN. (K) Quantitative analysis of M2 in tumor. (L) Flow cytometry detection of the proportion of Tregs in tumor. (M) Flow cytometry detection of the proportion of M1 in tumor. (N) Flow cytometry detection of the proportion of M2 in tumor. Data were given as mean ± SD. ** P < 0.01, *** P < 0.001, n = 3.

PD-1/PD-L1 immunotherapy has emerged as a promising modality for cancer treatment by inhibiting the PD-1/PD-L1 signaling pathway, thereby enabling the immune-mediated eradication of tumor cells through lymphocytic activity, including that of cytotoxic T lymphocytes, ultimately inducing cancer cell death 42. Despite its broad applicability across various tumor types, its efficacy against TNBC remains limited. Hence, we investigated whether US-activated RmPLH, which potentiates the cGAS/STING pathway, acts synergistically with PD-1 inhibition in TNBC. As delineated in Figure 8A, after subjecting 4T1 tumor-bearing mice to distinct treatment regimens, a PD-1 inhibitor was administered three times, followed by flow cytometric analysis of immune cell populations in the tumor and lymph nodes on the 14th day.

The proportion of CD8+ and CD4+ T cells was found to be significantly elevated in primary tumors compared to other control groups, with CD8+ T cells reaching 27.1% and CD4+ T cells reaching 44.2% (Figure 8B, E-F). CD8+CD62L+CD44+ T cells play a significant role in tumor immunity. These cells are terminally differentiated effector T cells with potent cytotoxicity that are capable of directly killing cancer cells.^ 42^ The central effector T cells were increased in the tumor-draining lymph nodes (TDLN) to 35.4% (Figure 8C and H). These results underscore the efficacy of PD1+RmPLH+US in stimulating the immune system and enhancing antitumor responses under US treatment. Subsequent quantification of dendritic cell (DC) maturation (CD80+86+) in lymph nodes revealed a substantial increase, particularly in the PD-1+US+RmPLH group, reaching approximately 54.7%, markedly surpassing other groups. These findings collectively suggest that US-activated RmPLH potentiates tumor infiltration by cytotoxic T cells and promotes DC maturation in the TDLN of 4T1 tumor-bearing mice, thereby enhancing the efficacy of PD-1 inhibitors in TNBC. Tregs play a pivotal antagonist role in tumor immunotherapy 43. In contrast to the other groups, PD1+RmPLH+US markedly reduced the infiltration of Tregs into the tumor region, thereby positively influencing tumor therapy. In the PD1+RmPLH+US group, Treg cells in the tumor infiltration had reduced to 6.94% (Figure 8L and G). Macrophage polarization within tumor regions plays a crucial role in the efficacy of immunotherapy. Macrophage polarization was quantified by marking CD11b+ cells with CD86+ for pro-immunogenic M1 macrophages and CD206+ for immunosuppressive M2 macrophages 44. According to the flow results, the proportion of M1 increased to 25.2% in the PD1+RmPLH+US group and the proportion of M2 decreased to 13.3% in the PD1+RmPLH+US group (Figure 8M, I, K, and N)**.** Taken together, these findings suggest that US-activated RmPLH enhances immunotherapeutic efficacy, thereby increasing the efficacy of PD-1 inhibitors against TNBC. The flow cytometric gating strategy for streaming is illustrated in Figure S15.

### Lung metastasis inhibition *via* the PD-1 inhibitor and RmPLH

**Figure 9:**
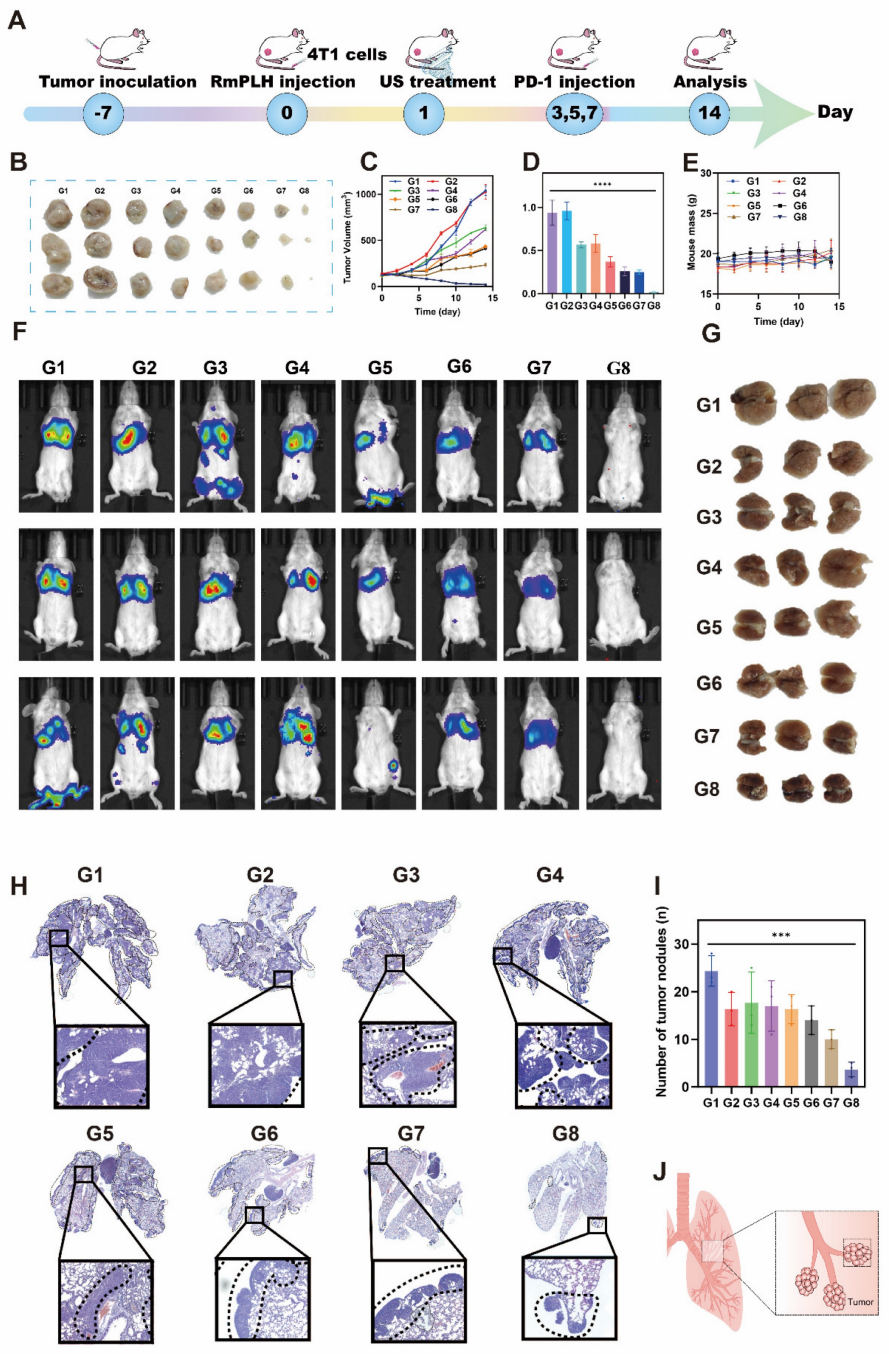
** Lung metastasis inhibition *via* the PD-1 and RmPLH.** (G1: PBS, G2:US, G3: PD-1, G4: PD-1+US, G5: PD-1+RmPLH, G6: PLH+US, G7: RmPLH+US, G8: PD-1+RmPLH+US) (A) Schematic depiction of the treatment plan for lung metastasis via the RmPLH. (B) Digital photo images of tumors in various groups. (C) Growth curves of tumors in different groups. (D) The weight of tumors at the end of treatment. (E) Body weight changes of the mice under different treatments. (F) *In vivo* fluorescence imaging of lung metastasis model for immunotherapy through RmPLH. (G) Representative digital photos of resected lung tissue in different treatment groups. (H) Representative images of H&E-stained lung tissues via various treatments. (I) The number of tumors nodlues in lung tissue. Data were given as mean ± SD. ** *P* < 0.01, *** *P* < 0.001, n = 3.

Lung metastasis is a common late-stage complication of TNBC. As depicted in Figure 5, the activation of RmPLH by US demonstrates the ability to attenuate the pseudopodia formation in 4T1 cells, consequently reducing their migratory and invasive properties, which represents a crucial step in preventing distant metastasis. Furthermore, the findings shown in Figure 8 underscore the ability of PD-1 inhibitors, in conjunction with US-activated RmPLH, to notably enhance the infiltration of cytotoxic T cells within tumor tissues facilitating DC maturation in 4T1 tumor-bearing mice, thereby improving the therapeutic efficacy of PD-1 inhibitors in TNBC. Motivated by these observations, we investigated whether the combined treatment of RmPLH-activated tumor tissue with US followed by the administration of a PD-1 inhibitor could reduce lung metastasis in murine models. In the experimental setup outlined in Figure 9A, 4T1 tumor-bearing mice injected with 4T1 cell suspension and RmPLH via the tail vein on day 0, followed by distinct treatments starting on day 1. PD-1 inhibitors were administered three times and lung metastasis in 4T1 tumor-bearing mice was assessed using a small animal fluorescence imaging system. Mice in the PD-1+RmPLH+US group exhibited significantly reduced tumor burdens (Figure 9B-D) compared to the other groups. On the 14th day, assessment via small animal fluorescence imaging revealed lung metastasis and tumor formation, as depicted in Figure 9J. Severe metastasis occurred in the control groups compared to that in the PD1+RmPLH+US group (Figure 9F). Further examination of lung tissues revealed that PD1+RmPLH+US significantly suppressed lung metastasis very significantly, while all other groups exhibited varying degrees of metastasis (Figure 9G). This conclusion was further supported by histopathological analysis of tissue sections (Figure 9H). Statistical analysis of tumor nodules, indicated a significant decrease in the number of nodules in the PD1+RmPLH+US group compared to other groups (Figure 9I). RmPLH-activated tumor tissue with US and PD-1 inhibitors significantly reduced lung metastasis in a mouse model of TNBC.

## Conclusions

The persistent challenges in effectively treating TNBC underscore the critical need for innovative therapeutic approaches. Addressing this urgent clinical demand, we propose an advanced hollow nanostructured drug delivery system, termed RmPLH, which harnesses the unique properties of red blood cell encapsulation to prolong drug circulation and enhance therapeutic efficacy. Our investigation demonstrates that Pt (II)-CoNi LDH generates ROS under ultrasound stimulation after cellular uptake. This ROS surge inflicts substantial intracellular damage, induces ER stress, and activates the cGAS/STING signaling pathway. This activation elicits ICD, hallmarked by pyroptosis and ferroptosis. Notably, the cascade of events triggered by RmPLH significantly potentiates therapeutic outcomes and achieves synergistic efficacy when combined with PD-1 checkpoint inhibitors. These findings present a novel and promising therapeutic strategy for the effective management of TNBC, potentially paving the way for transformative advancements in overcoming this formidable oncological challenge.

## Supplementary Material

Supplementary figures and tables.

## Abbreviations

cGAS: Cyclic GMP-AMP synthase
CLSM: Confocal laser scanning microscopy
CRT: Calreticulin
DAMPs: Danger-associated molecular patterns
ER: Estrogen receptor
F-actin: Filamentous actin
γ-H2AX: Gamma H2AX
GPX4: Glutathione peroxidase 4
GSDME: Gasdermin E
GSH: Glutathione
HER2: Human epidermal growth factor receptor 2
HMGB1: High mobility group box 1
ICD: Immunogenic cell death
LC3 II: Microtubule-associated protein 1A/1B-light chain 3
LDHs: Layered double hydroxides
MDA: Malondialdehyde
MOFs: Metal-organic
PD-1: Programmed cell death protein 1
PR: Progesterone receptor
RBCm@Pt-CoNi LDH (RmPLH): Red blood cell membrane-coated Pt-CoNi layered double hydroxide
ROS: Reactive oxygen species
SDT: Sonodynamic therapy
STING: Stimulator of interferon genes
TAC: Total antioxidant capacity
TDLN: Tumor-draining lymph nodes
Tregs: Regulatory T cells
TNBC: Triple-negative breast cancer
XBP1s: Spliced X-box binding protein 1

## References

[1] Derakhshan F, Reis-Filho JS (2022). Pathogenesis of triple-negative breast cancer. Annu Rev Pathol.

[2] Yin L, Duan J-J, Bian X-W, Yu S-c (2020). Triple-negative breast cancer molecular subtyping and treatment progress. Breast Cancer Res.

[3] Li Y, Wang C, Xu T, Pan P, Yu Q, Xu L (2021). Discovery of a small molecule inhibitor of cullin neddylation that triggers ER stress to induce autophagy. Acta Pharm Sin B.

[4] Marshall SK, Angsantikul P, Pang Z, Nasongkla N, Hussen RSD, Thamphiwatana SD (2022). Biomimetic targeted theranostic nanoparticles for breast cancer treatment. Molecules.

[5] Brenner JS, Mitragotri S, Muzykantov VR (2021). red blood cell hitchhiking: a novel approach for vascular delivery of nanocarriers. Annu. Rev. Biomed. Eng.

[6] Liang JY, Zhang XD, Zeng XX, Yan M, Yin YX, Xin S (2020). Enabling a durable electrochemical interface via an artificial amorphous cathode electrolyte interphase for hybrid solid/Liquid lithium-metal batteries. Angew Chem Int Ed Engl.

[7] Zhang X, Huang Y, Yang X (2021). The complex role of PD-L1 in antitumor immunity: a recent update. Cell Mol Immunol.

[8] Sprooten J, Laureano RS, Vanmeerbeek I, Govaerts J, Naulaerts S, Borras DM (2023). Trial watch: chemotherapy-induced immunogenic cell death in oncology. OncoImmunology.

[9] Xie J, Yu F, Zhao J, Guo W, Zhang H-L, Cui G (2020). An irreversible electrolyte anion-doping strategy toward a superior aqueous Zn-organic battery. Energy Stor Mater.

[10] Han X, Wei Q, Lv Y, Weng L, Huang H, Wei Q (2022). Ginseng-derived nanoparticles potentiate immune checkpoint antibody efficacy by reprogramming the cold tumor microenvironment. Mol Ther.

[11] Hu T, Gu Z, Williams GR, Strimaite M, Zha J, Zhou Z (2022). Layered double hydroxide-based nanomaterials for biomedical applications. Chem Soc Rev.

[12] Zhang C, Shao M, Zhou L, Li Z, Xiao K, Wei M (2016). Hierarchical NiFe layered double hydroxide hollow microspheres with highly-efficient behavior toward oxygen evolution reaction. ACS Appl Mater Interfaces.

[13] Qin Y, Wang B, Qiu Y, Liu X, Qi G, Zhang S (2021). Multi-shelled hollow layered double hydroxides with enhanced performance for the oxygen evolution reaction. Chem Commun (Camb).

[14] Pan Y, Lin R, Chen Y, Liu S, Zhu W, Cao X (2018). Design of single-atom Co-N5 catalytic site: a robust electrocatalyst for CO2 reduction with nearly 100% CO selectivity and remarkable stability. J Am Chem Soc.

[15] Deng Z, Wang N, Liu Y, Xu Z, Wang Z, Lau T-C (2020). A photocaged, water-oxidizing, and nucleolus-targeted Pt(IV) complex with a distinct anticancer mechanism. J Am Chem Soc.

[16] Shan J, Liao J, Ye C, Dong J, Zheng Y, Qiao SZ (2022). The dynamic formation from metal-organic frameworks of high-density platinum single-atom catalysts with metal-metal interactions. Angew Chem Int Ed Engl.

[17] Liang G, Sadhukhan T, Banerjee S, Tang D, Zhang H, Cui M (2023). Reduction of Platinum(IV) prodrug hemoglobin nanoparticles with deeply penetrating ultrasound radiation for tumor-targeted therapeutically enhanced anticancer therapy. Angew Chem Int Ed Engl.

[18] Hu T, Shen W, Meng F, Yang S, Yu S, Li H (2023). Boosting the sonodynamic cancer therapy performance of 2D layered double hydroxide nanosheet-based sonosensitizers via crystalline-to-amorphous phase transformation. Adv Mater.

[19] Tham MJR, Babak MV, Ang WH (2020). PlatinER: a highly potent anticancer platinum(II) complex that induces endoplasmic reticulum stress driven immunogenic cell death. Angew Chem Int Ed Engl.

[20] Han S, Li H, Li T, Chen F, Yang R, Yu Y (2023). Ultralow overpotential nitrate reduction to ammonia via a three-step relay mechanism. Nat Catal.

[21] Wang J, Huang Z, Liu W, Chang C, Tang H, Li Z (2017). Design of N-coordinated dual-metal sites: a stable and active Pt-free catalyst for acidic oxygen reduction reaction. J Am Chem Soc.

[22] Li Y, Jin Z, Zhao T (2020). Performance of ZIF-67 - derived fold polyhedrons for enhanced photocatalytic hydrogen evolution. Chem Eng J.

[23] Pan Y, Zhang C, Lin Y, Liu Z, Wang M, Chen C (2020). Electrocatalyst engineering and structure-activity relationship in hydrogen evolution reaction: from nanostructures to single atoms. Sci China Mater.

[24] Yu D, Wu B, Ge L, Wu L, Wang H, Xu T (2016). Decorating nanoporous ZIF-67-derived NiCo^2^O^4^ shells on a Co^3^O^4^ nanowire array core for battery-type electrodes with enhanced energy storage performance. Journal of Materials Chemistry A.

[25] Göksu H, Zengin N, Burhan H, Cellat K, Şen F (2020). A novel hydrogenation of nitroarene compounds with multi wall carbon nanotube supported palladium/copper nanoparticles (PdCu@MWCNT NPs) in aqueous medium. Sci Rep.

[26] Chikte S, Panchal N, Warnes G (2013). Use of lysotracker dyes: a flow cytometric study of autophagy. Cytometry A.

[27] Aranda A, Sequedo L, Tolosa L, Quintas G, Burello E, Castell JV (2013). Dichloro-dihydro-fluorescein diacetate (DCFH-DA) assay: A quantitative method for oxidative stress assessment of nanoparticle-treated cells. Toxicol In Vitro.

[28] Guthrie HD, Welch GR, Long JA (2008). Mitochondrial function and reactive oxygen species action in relation to boar motility. Theriogenology.

[29] Robichaux DJ, Harata M, Murphy E, Karch J (2023). Mitochondrial permeability transition pore-dependent necrosis. J Mol Cell Cardiol.

[30] De Miranda BR, Rocha EM, Castro SL, Greenamyre JT (2020). Protection from α-synuclein induced dopaminergic neurodegeneration by overexpression of the mitochondrial import receptor TOM20. NPJ Parkinsons Dis.

[31] Zhou B, Zhang J-y, Liu X-s, Chen H-z, Ai Y-l, Cheng K (2018). Tom20 senses iron-activated ROS signaling to promote melanoma cell pyroptosis. Cell Res.

[32] Gaschler MM, Stockwell BR (2017). Lipid peroxidation in cell death. Biochem Biophys Res Commun.

[33] Liu Y, Wan Y, Jiang Y, Zhang L, Cheng W (2023). GPX4: The hub of lipid oxidation, ferroptosis, disease and treatment. Biochim Biophys Acta Rev Cancer.

[34] Chen GY, Nuñez G (2010). Sterile inflammation: sensing and reacting to damage. Nat Rev Immunol.

[35] Bruno PM, Liu Y, Park GY, Murai J, Koch CE, Eisen TJ (2017). A subset of platinum-containing chemotherapeutic agents kills cells by inducing ribosome biogenesis stress. Nat Med.

[36] Galluzzi L, Vitale I, Aaronson SA, Abrams JM, Adam D, Agostinis P (2018). Molecular mechanisms of cell death: recommendations of the Nomenclature Committee on Cell Death 2018. Cell Death Differ.

[37] Chen S, Chen J, Hua X, Sun Y, Cui R, Sha J (2020). The emerging role of XBP1 in cancer. Biomed Pharmacother.

[38] Dobbs N, Burnaevskiy N, Chen D, Gonugunta Vijay K, Alto Neal M, Yan N (2015). STING activation by translocation from the ER Is associated with infection and autoinflammatory disease. Cell Host Microbe.

[39] Jiménez-Loygorri JI, Villarejo-Zori B, Viedma-Poyatos Á, Zapata-Muñoz J, Benítez-Fernández R, Frutos-Lisón MD (2024). Mitophagy curtails cytosolic mtDNA-dependent activation of cGAS/STING inflammation during aging. Nat Commun.

[40] Zhao H, Wu L, Yan G, Chen Y, Zhou M, Wu Y (2021). Inflammation and tumor progression: signaling pathways and targeted intervention. Signal Transduct Target Ther.

[41] Yamaguchi H, Condeelis J (2007). Regulation of the actin cytoskeleton in cancer cell migration and invasion. Biochim Biophys Acta Mol Cell Res.

[42] Parvez A, Choudhary F, Mudgal P, Khan R, Qureshi KA, Farooqi H (2023). PD-1 and PD-L1: architects of immune symphony and immunotherapy breakthroughs in cancer treatment. Front Immunol.

[43] Li C, Jiang P, Wei S, Xu X, Wang J (2020). Regulatory T cells in tumor microenvironment: new mechanisms, potential therapeutic strategies and future prospects. Mol Cancer.

[44] Chen S, Saeed A, Liu Q, Jiang Q, Xu H, Xiao GG (2023). Macrophages in immunoregulation and therapeutics. Signal Transduct Target Ther.

