# Supplementary material for: Ultrasound-activated erythrocyte membrane-camouflaged Pt (II) layered double hydroxide enhances PD-1 inhibitor efficacy in triple-negative breast cancer through cGAS-STING pathway-mediated immunogenic cell death
Source: Theranostics. 2025 Jan 2;15(4):1456–77. doi: 10.7150/thno.102284 (PMC11729553; doi:10.7150/thno.102284)
Supplement: Supplementary file 1 — Supplementary figures and tables. [file thnov15p1456s1.pdf]

Supporting Information

Ultrasound-activated erythrocyte membrane-camouflaged Pt (II) layered doubl hydroxide enhances PD-1 inhibitor efficacy in triple-negative breast cancer through cGAS-STING pathway-mediated immunogenic cell death

Yanjie Wu[2#], Zhiyu Zhao[1,3#], Mengli Ma[1#], Weijin Zhang[1], Wei Liu[4], Xiaochen Liang[5], Ting Zhao[5], Yi Luo[6], Yunjie Wang[1], Mengqi Li[1], Tingting Li[8], Cong Liu[8], Xian Luo[1], Shengyu Wang[1], Wanyun Li[1], Wei Zeng[7], Hong Wang[7], Wengang Li[1], Ting Wu[1*], Zhihai Ke[2]*, Fanghong Luo[1]*

1. Cancer Research Center, School of Medicine, Xiamen University, Xiamen, 3 61102, P. R. China

2. School of Science and Engineering, Shenzhen Key Laboratory of Innovative Drug Synthesis, The Chinese University of Hong Kong, Shenzhen, 518172, P. R. China

3. Thomas Lord Department of Mechanical Engineering and Materials Science, Duke University, Durham, NC, 27708, USA

4. The First Affiliated Hospital of Guangdong Pharmaceutical University, Guan gdong Pharmaceutical University, Guangzhou, 510026, P. R. China

5. Environmental Toxicology, University of California, Riverside, CA, 92507, U SA

6. Institute of Immunotherapy, School of Basic Medicine, Fujian Medical Univ ersity, Fuzhou, 350122, P. R. China

7. Department of Gastroenterology, Department of Obstetrics and Gynecology, Affiliated Xiang'an Hospital, Medical Center, Xiamen University, 361102, P. R. China

8. Department of Anesthesiology, Shandong Provincial Hospital Affiliated to Sh andong First Medical University, Jinan, Shandong, 250021, China

#These authors have contributed equally to this work

Corresponding authors: Ting Wu (wuting78@189.cn); Zhihai Ke (kezhihai@cuhk.edu.cn); Fanghong Luo (luofanghong@xmu.edu.cn).

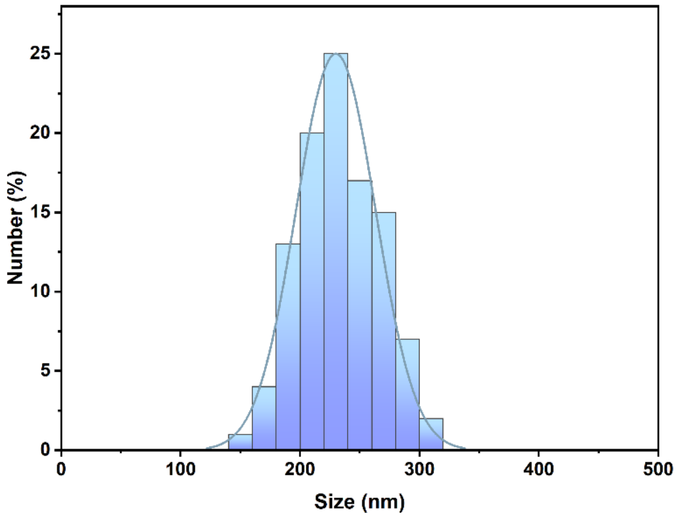

Figure S1. Particle size values of PLH.

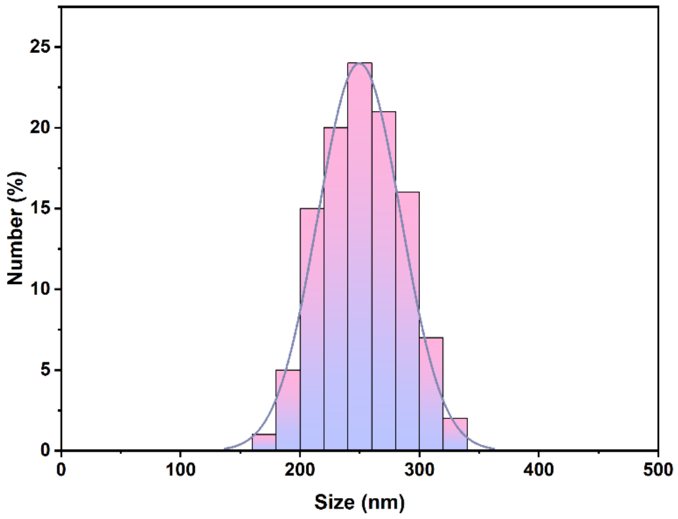

Figure S2. Particle size values of RmPLH.

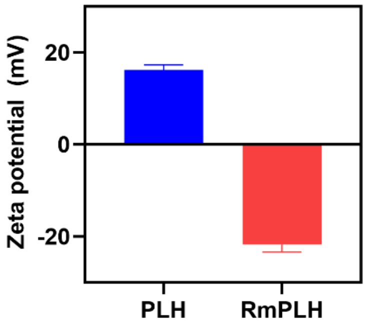

Figure S3. Zeta potential values of PLH and RmPLH.

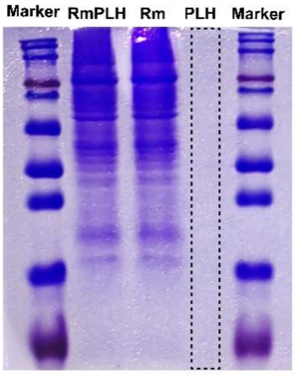

Figure S4. SDS-PAGE electrophoresis of Rm, PLH and RmPLH.

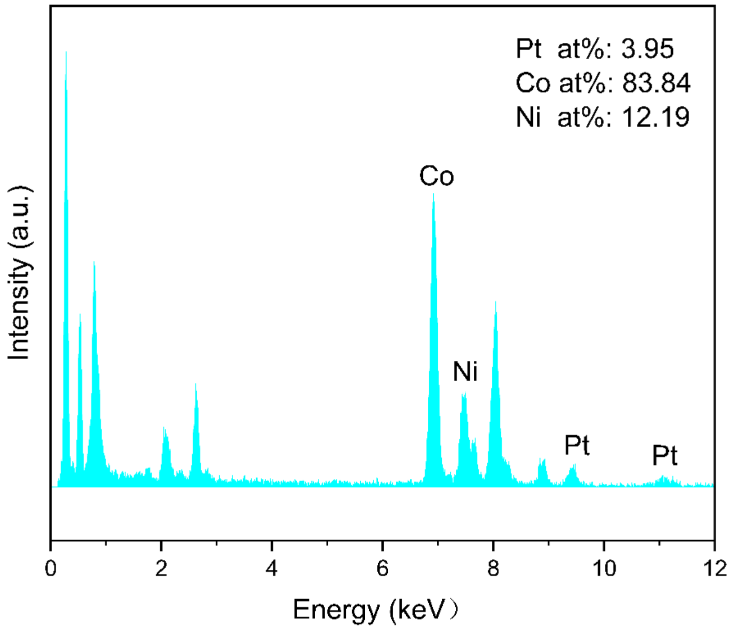

Figure S5. EDS spectrum of Pt-CoNi LDH. Inset, the atomic percentages of Pt, Co and Ni in the Pt-CoNi LDH estimated from the EDS.

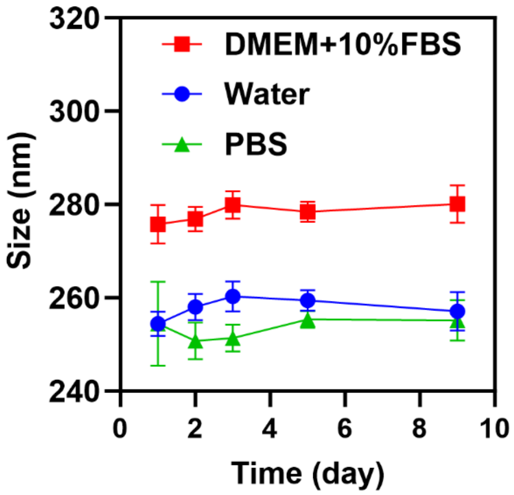

Figure S6. Particle size analysis of RmPLH in water, PBS and cell culture medium at different time points.

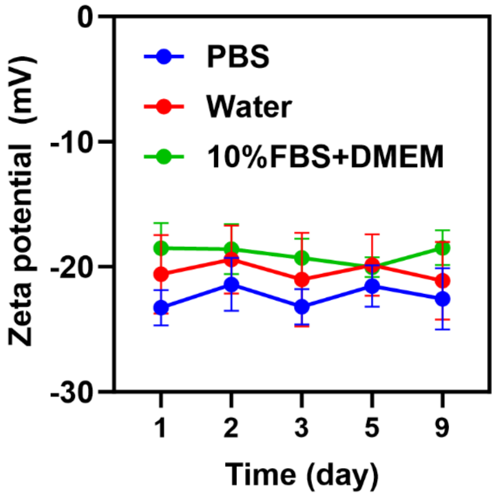

Figure S7. Zeta potential analysis of RmPLH in water, PBS and cell culture medium at different time points.

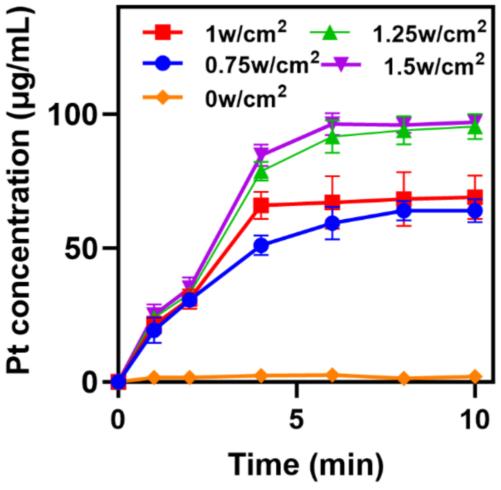

Figure S8.ICP-MS Determination of Pt released by ultrasonication at different powers and times.

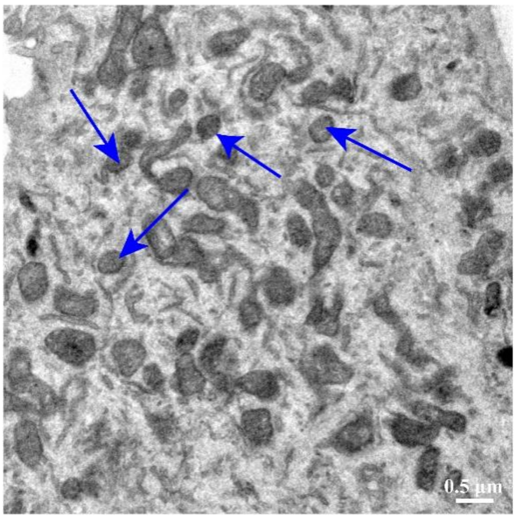

Figure S9. Ferroptosis mitochondrial lesions after RmPLH+US treatment (blue arrow).

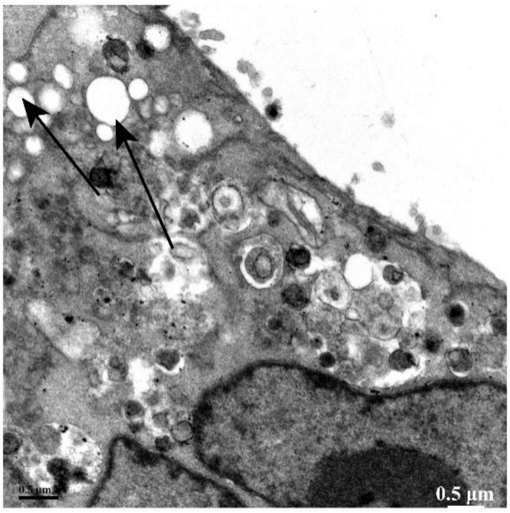

Figure S10. TEM of the formation of numerous vesicles in cells treated with RmPLH+US. (Black arrow)

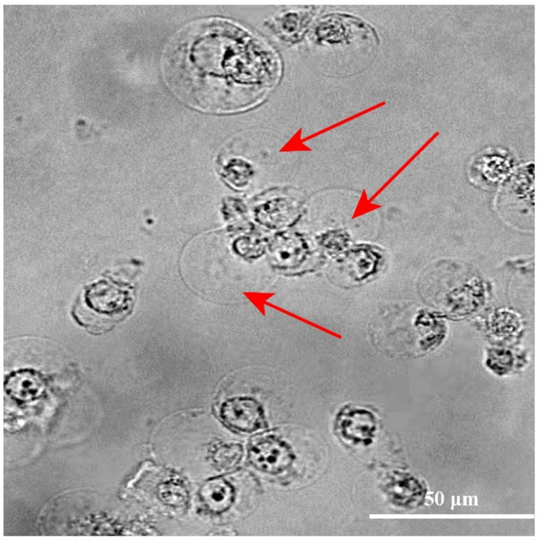

Figure S11. Morphology of cellular pyroptosis under RmPLH+US treatment (red arrow).

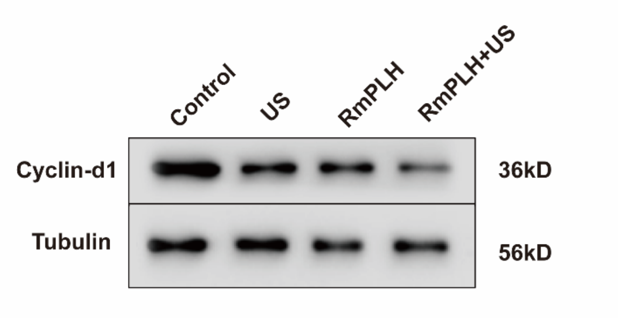

Figure S12. Expression of Cyclin-d1 in 4t1 cells after different treatments.

Table S1. The weight of the mice

Markers<br>Weight (g)<br>Time	DAY 0	DAY 2	DAY 4	DAY 6	DAY 8	DAY 10	DAY 12	DAY 14
(I)-1	20	20.6	20.5	20.1	20.9	20.7	20.4	20.2
(I)-2	20.7	20	20.8	20.9	20.6	20	20.9	20.4
(I)-3	20.5	20.6	20.4	20.9	20.7	20.7	20.4	20.6
(I)-4	20.8	20.9	20.8	20.6	20.5	20.9	20	20.7
(I)-5	20	20.7	20.4	20.7	20.6	20.8	20	20.4
(II)-1	20	20.6	20.5	20.1	20.9	20.7	20.4	20.2
(II)-2	20.7	20	20.8	20.9	20.6	20	20.9	20.4

(Ⅱ)-3	20.5	20.6	20.4	20.9	20.7	20.7	20.4	20.6
(Ⅱ)-4	20.8	20.9	20.8	20.6	20.5	20.9	20	20.7
(Ⅱ)-5	20	20.7	20.4	20.7	20.6	20.8	20	20.4
(Ⅲ)-1	20.9	21.7	20.7	20.4	20.4	21.5	20.1	21.4
(Ⅲ)-2	20.1	20	20.9	20.1	21	20	20.9	20.3
(Ⅲ)-3	21.3	21.4	20.7	20.9	21.3	20.7	20.8	20.7
(Ⅲ)-4	21.4	20	20.5	20.3	20.3	20.7	20.5	20.7
(Ⅲ)-5	20.6	20.6	21	21.9	21.7	20.1	21.3	20.4
(Ⅳ)-1	21.4	20.9	21	20.5	21.1	20.9	21.5	21.4
(Ⅳ)-2	20.1	20.8	20.3	21.7	21.3	21.8	21.1	20.8
(Ⅳ)-3	20.6	21.7	20.1	20.8	20.4	21.4	20.9	20.7
(Ⅳ)-4	20.7	20.1	21.9	21.5	20.8	21.3	21.4	20
(Ⅳ)-5	20.8	21.3	21.9	20	21.4	21.5	21.5	21

BALB/C tumor-bearing mice were randomly divided into four groups:

(ⅰ) PBS, (ⅱ) US, (ⅲ) RmPLH, (ⅳ) RmPLH+US.

Table S2. Biochemical blood analysis of the mice treated after different treatments

Markers	WBC (10^9/L)	RBC (10^12/L)	HGB (g/L)	PLT (10^11/L)	MCV (fL)	MCH (pg)	MCHC (g/L)
(Ⅰ)-1	8.1	8.76	147	13.59	45	16.7	373
(Ⅰ)-2	6.4	8.51	143	10.41	48.2	16.8	348
(Ⅰ)-3	6.5	8.9	142	10.17	47.3	15.9	338
(Ⅰ)-4	7.2	8.24	141	9.69	49	17.1	349
(Ⅰ)-5	2.5	8.05	141	11.44	47.4	17.5	370
(Ⅱ)-1	4.2	8.54	143	10.5	48.2	16.7	347

(Ⅱ)-2	11.7	8.69	136	11.32	47.1	15.6	332
(Ⅱ)-3	6.1	8.16	138	10.21	48.8	16.9	346
(Ⅱ)-4	6.4	8.29	143	10.58	48.6	17.2	355
(Ⅱ)-5	6.3	8	140	13.58	47.9	17.5	365
(Ⅲ)-1	7.2	8.69	146	10.71	48.6	16.8	345
(Ⅲ)-2	7.2	7.57	136	8.16	47.9	17.9	375
(Ⅲ)-3	5.4	8.92	146	8.2	46.5	16.3	352
(Ⅲ)-4	8.5	9.13	146	11.02	48.1	15.9	332
(Ⅲ)-5	4.9	8.96	137	16.02	46.6	15.2	328
(Ⅳ)-1	9.5	8.95	141	11.66	47.6	15.7	330
(Ⅳ)-2	6.5	8.19	130	18.03	47.2	15.8	336
(Ⅳ)-3	2.3	8.04	134	12.09	47.7	16.6	349
(Ⅳ)-4	4.9	8.84	144	10.91	46.9	16.2	347
(Ⅳ)-5	4.4	8.9	135	26.46	50.4	15.1	301

BALB/C tumor-bearing mice were randomly divided into four groups:

(ⅰ) PBS, (ⅱ) US, (ⅲ) RmPLH, (ⅳ) RmPLH+US.

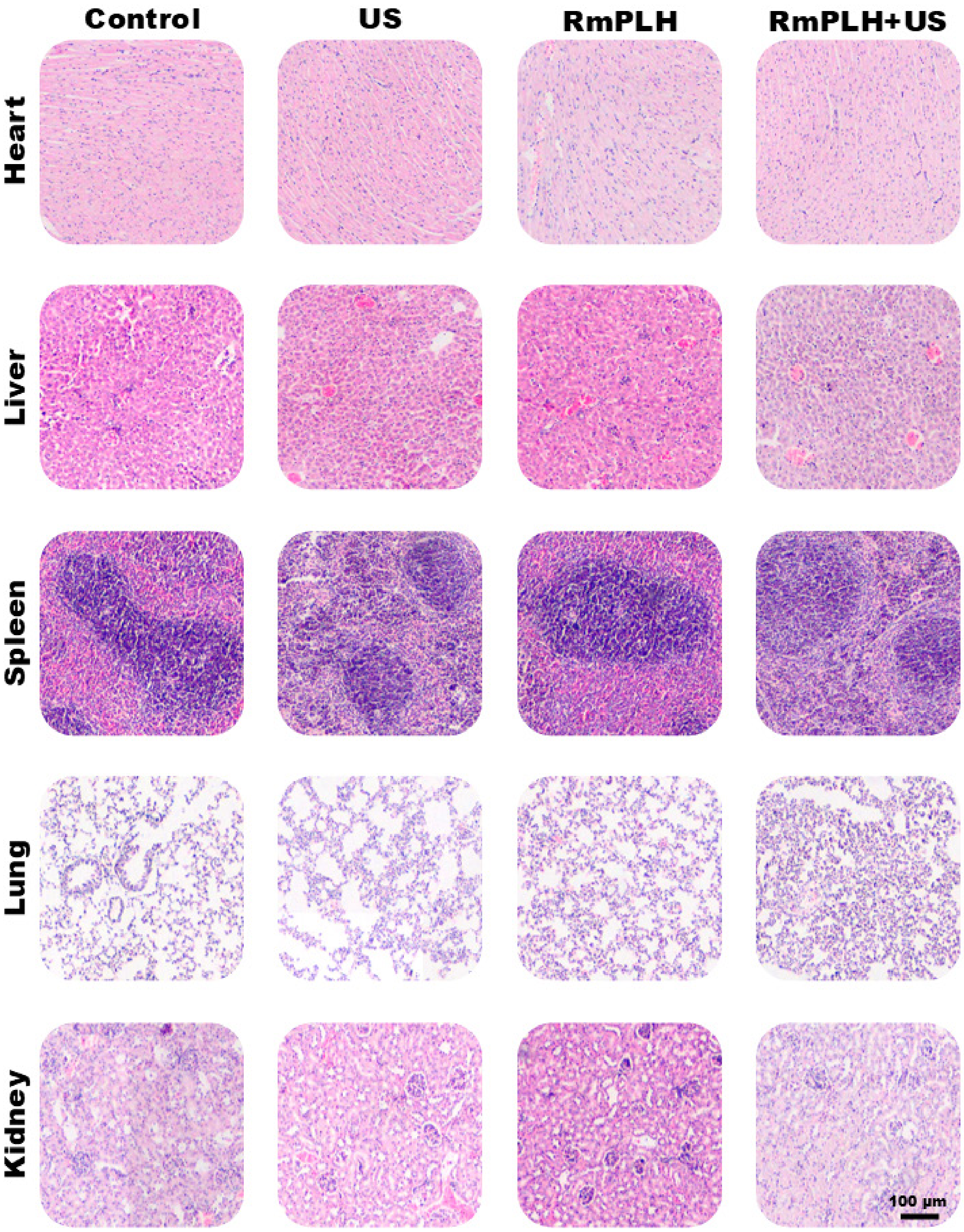

Figure S13. H&E staining of normal tissues from different treatment group.

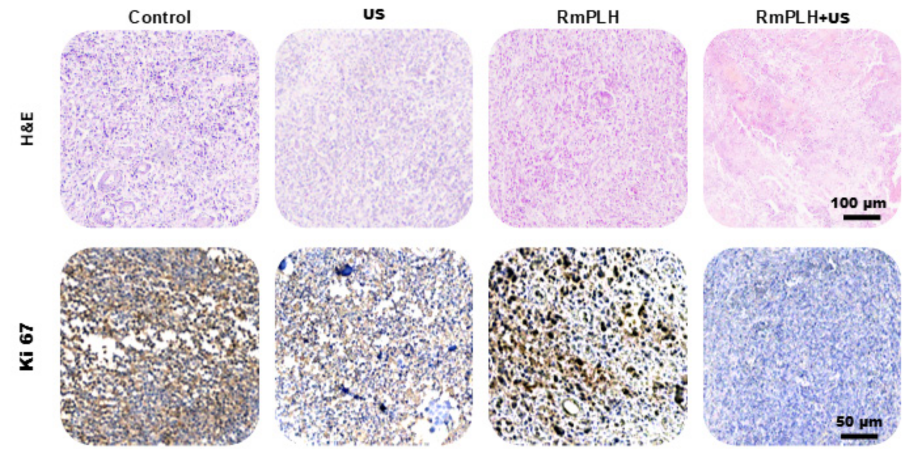

Figure S14. Images demonstrate H&E staining and Ki-67 immunohistochemistry of tumors collected on the 14th of different therapies.

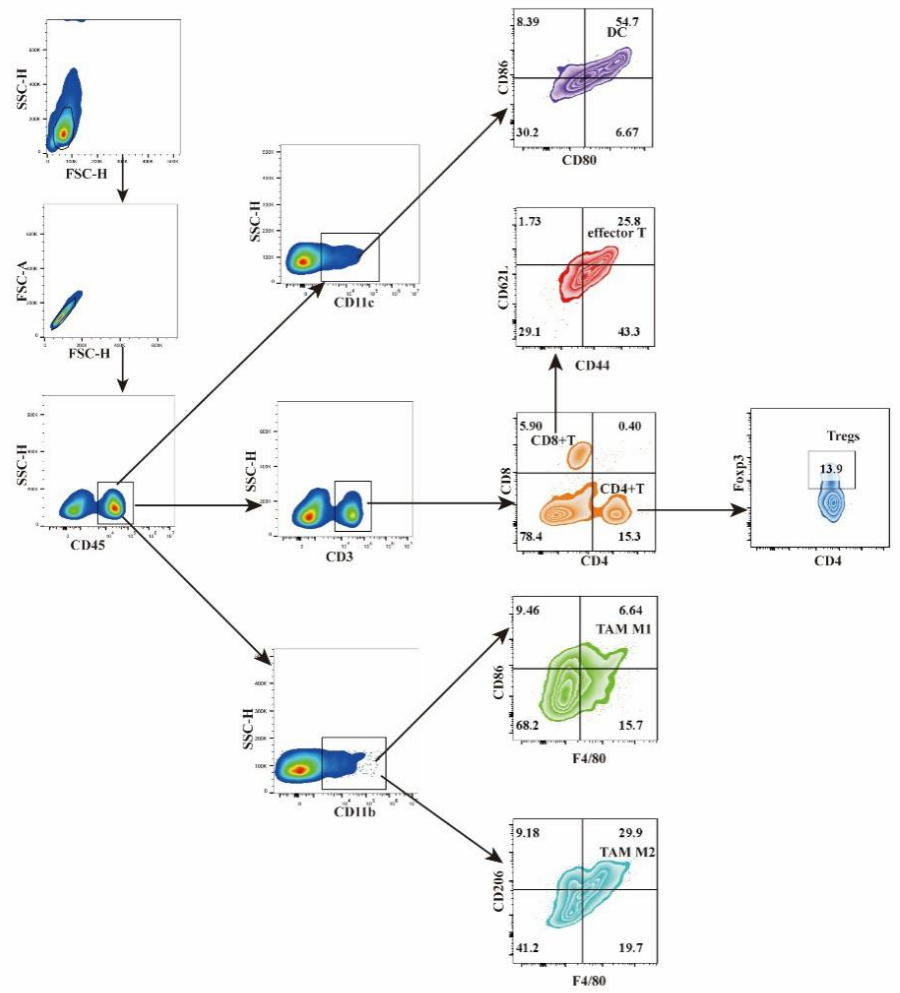

Figure S15. Gating strategies for the analysis of mature DCs (CD45+CD11c+CD80CD86+), CD4+T cells (CD45+CD3+CD4+), CD8+T cells (CD45+CD3+CD8+) Treg (CD45+CD3+CD4+Foxp3+), Tcm (CD45+CD3+CD8+CD44+CD62L+), TAM M1 (CD45+CD11b+F4/80+CD86+) and TAM M2 (CD45+CD11b+F4/80+CD206+) *in vivo* by flow cytometry.

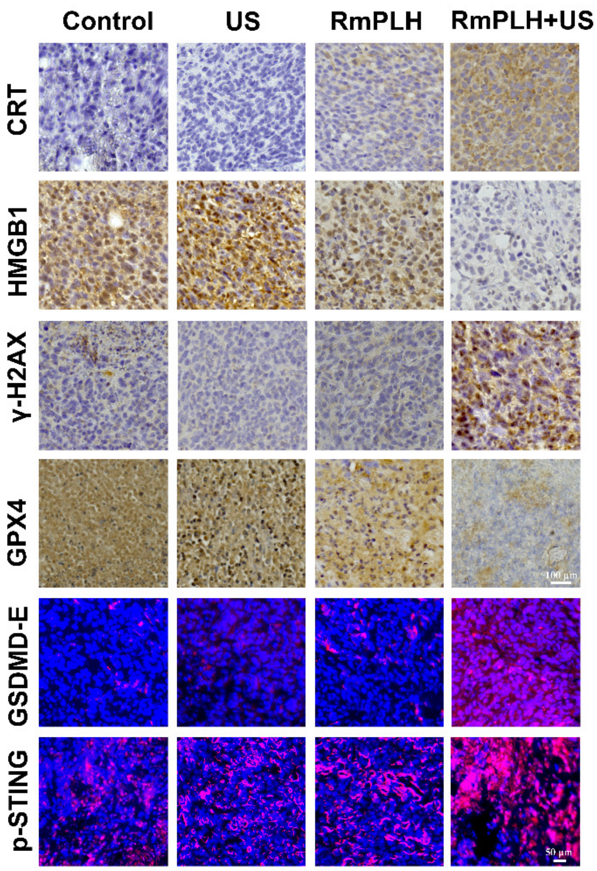

Figure S16. The expressions of CRT, HMGB1, γ-H2AX, GPX4, GSDME-N and p-STING in 4T1 tumor tissues after different treatments were detected by immunohistochemistry and immunohistofluorescence.
